# Non-Pharmaceutical Interventions for Self-Regulatory Failures in Adolescents Suffering from Externalizing Symptoms: A Scoping Review

**DOI:** 10.3390/biomedicines9091081

**Published:** 2021-08-24

**Authors:** Lauriane Constanty, Caroline Lepage, Joëlle Rosselet Amoussou, Emilie Wouters, Velia Decoro, Lisa De-Paz, Charlotte Hans, Hazal Ergüneş, Jonas Sangra, Kerstin Jessica Plessen, Sébastien Urben

**Affiliations:** 1 Division of Child and Adolescent Psychiatry, Lausanne University Hospital (CHUV), University of Lausanne, 1004 Lausanne, Switzerland; lauriane.constanty@chuv.ch (L.C.); Caroline.Lepage@chuv.ch (C.L.); v.decoro@gmail.com (V.D.); l.depaz8@gmail.com (L.D.-P.); charlotte_hans@outlook.com (C.H.); hazal.ergunes@gmail.com (H.E.); Jonas.Sangra@unil.ch (J.S.); kerstin.plessen@chuv.ch (K.J.P.); 2 Psychiatry Library, Education and Research Department, Lausanne University Hospital, University of Lausanne, 1008 Prilly, Switzerland; Joelle.Rosselet@chuv.ch; 3 Unit of Child and Adolescent Forensic Psychiatry, Lausanne University Hospital (CHUV), 1008 Prilly, Switzerland; Emilie.Wouters@chuv.ch

**Keywords:** self-regulatory processes, non-pharmaceutical interventions, externalizing symptoms, network analysis, adolescents, study design, intervention stage

## Abstract

Introduction: Deficits of self-regulation (SR) are a hallmark of externalizing (EXT: offending or aggressive behaviors) symptoms in adolescence. Objectives: This scoping review aims (1) to map non-pharmaceutical interventions targeting SR processes to reduce EXT symptoms in adolescents and (2) to identify research gaps, both of which will provide recommendations for future studies. Methods: Systematic searches were carried out in eight bibliographic databases up to March 2021, combining the following concepts: self-regulation, externalizing symptoms, adolescents, and non-pharmaceutical interventions. Results: We identified 239 studies, including 24,180 youths, mainly from North America, which described a plethora of non-pharmaceutical interventions targeting SR to alleviate EXT symptoms in adolescents (10–18 years of age). The majority of studies (about 70%, k = 162) represent samples with interventions exposed to “selective” or “indicated” prevention. Curriculum-based (i.e., multiple approaches targeting several domains such as emotion, cognition, and social) interventions (31.4%) were the most common type of intervention. Moreover, studies on cognitive-based interventions, mind-based interventions, and emotional-based interventions have increased over the last decades. Network analyses allowed us to identify several hubs between curriculum-based interventions, cognitive SR processes, as well as aggressiveness, conduct problems, and irritability/anger dysregulation. In addition, we identified gaps of studies concerning the physiological SR processes and on some types of interventions (i.e., body-based interventions and externally mediated interventions) or, more specifically, on promising tools, such as biofeedback, neurofeedback, as well as programs targeting neuropsychological processes (e.g., cognitive remediation). Conclusions: This scoping review stresses the plethora of interventions, identified hubs, and emerging fields, as well as some gaps in the literature, which together may orient future studies.

## 1. Introduction

Externalizing (EXT) symptoms (e.g., disruptive behaviors, aggression, or rule-breaking behaviors) in adolescence represent a major public health concern. Indeed, EXT symptoms may lead to serious human (e.g., individuals must deal with his disruptive behavior), social (e.g., perception of insecurity and victimization), and economic (e.g., prevention, treatments, and trials) costs [1,2,3]. For some individuals, destructive and delinquent behaviors reach a peak at adolescence and gradually decrease into adulthood, and for others, these behaviors may be more profoundly anchored in functioning, initiating a long-term criminal development [4,5,6,7,8,9,10]. Identifying risk factors that may lead to a chronic trajectory is thus an important field of investigation in forensic psychiatry. In this perspective, self-regulatory (SR) difficulties are a hallmark of EXT symptoms, leading to offending and/or destructive behaviors e.g., [11,12,13,14]. Many tools or programs that specifically target SR deficits to reduce EXT symptoms in adolescents have been developed and examined in previous studies. Within this context, the aims of this scoping review were to map the non-pharmaceutical interventions targeting SR processes to reduce EXT symptoms in adolescents and to identify gaps to provide recommendations for the research agenda.

### 1.1. Externalizing Symptoms

Childhood EXT disorders have been associated with a higher risk of experiencing school failure, social difficulties, or future criminal activities, e.g., [9,15], as well as behavioral and emotional difficulties later in life, e.g., [9,10]. In particular, EXT behaviors may lead to important distress among adolescents and their surroundings [16]. According to the Diagnostic and Statistical Manual-5 [17], three main diagnoses encompassed EXT symptoms, namely the oppositional defiant disorder (ODD), the intermittent explosive disorder (IED), and the conduct disorder (CD). However, this categorical classification has several limitations, such as (a) considering psychopathologies as discrete categories, although they exist on a continuum from normal to pathological mental functioning, (b) limited reliability of the diagnoses, and (c) the neglect of subthreshold difficulties that generate suffering. Therefore, alternative visions to this classification have been proposed by the Hierarchical Taxonomy of Psychopathology (HiTOP) [18] or the Research Domain Criteria (RDoC) [19], which refer to dimensional approaches of psychopathologies. Hence, our scoping review adopted a dimensional perspective and focused on EXT symptoms (e.g., offending, aggressiveness or conduct problems). In this perspective, notice that some EXT symptoms such as aggression range from the normal range of behaviors (e.g., defensive aggression) to pathological ones (e.g., premeditated aggression), e.g., [20].

### 1.2. Self-Regulation Processes

SR refers to every process allowing the individuals to adapt their emotions, their thoughts, and their behaviors to the ever-changing environment and/or to achieve long-term goals [21]. Several definitions of SR exist in the literature, and this term may be used interchangeably with executive functions, effortful control, emotional regulation, or self-control, among others. More specifically, SR refers to the intrinsic psychological, biological, and social processes [21]. The psychological component of SR includes cognitive (e.g., effortful processes, executive functions), as well as emotional (e.g., emotion regulation) processes. The biological or physiological aspects of SR encompass the regulation of the autonomous nervous system and the hypothalamic–pituitary–adrenal (HPA) axis, which sustain the adjustment of physiological states to adapt to a context [21]. Finally, the social SR processes promote social affiliation and allow individuals to use appropriate facial expressions and adequate social communication in social situations [22].

### 1.3. SR Failures and EXT Symptoms

Many studies showed that deficits in SR are the main factors leading to impulsive behaviors and criminal activities [11,12,13]. Indeed, individuals with low SR processes tend to be impulsive, seeking risk and immediate gratification, and, thus, have a higher propensity to engage in offending behaviors (e.g., vandalism, petty theft). Moreover, low self-control, observed in low tolerance for frustration, may be the consequence of ineffective socialization and seems to be a stable trait throughout life [23]. More specifically, when psychological SR processes breakdown, leading to different dysfunctions, such as response disinhibition, impulsivity, or risk-taking [21]. Moreover, anger dysregulation (i.e., emotional SR processes deficits) is closely linked to more severe and persistent delinquent or violent behaviors e.g., [10,24,25,26,27]. Likewise, according to the polyvagal theory e.g., [22], deficits in the physiological SR processes may lead individuals to present emotional dysregulation and behavioral problems.

### 1.4. Adolescence

EXT symptoms reach a peak at adolescence [5,9], which may be related to the evaluation of risk that seems to be biased during adolescence e.g., [28], driven by the minimization of possible negative consequences and the maximization of immediate pleasure and gratification. Adolescence is, thus, a period of heightened sensation-seeking leading to substance use, sexual risky behavior, and other problematic and/or challenging behaviors such as EXT symptoms [29]. Moreover, during adolescence, the subcortical brain structures, sustaining emotional reactivity, are developed earlier, whereas the prefrontal cortex, allowing emotion regulation, continues their maturation until later, leading to a gap of controlling emotions and impulses during that period of development [30]. This situation leads to an “emotional overshoot”, which explains emotional over-reactivity, as well as accident-proneness and risk-taking in adolescents [30]. The initiation of interventions during this crucial period of brain plasticity offers a unique window of opportunity [31].

### 1.5. The Current Scoping Review

Some pharmacological interventions might be used to reduce EXT symptoms (for an umbrella review see [32]); however, due to important adverse effects of the most used agents in young people, the risk-benefit should be carefully assessed [33]. We, thus, undertook a scoping review to map the non-pharmaceutical interventions targeting SR processes aiming at reducing EXT symptoms in adolescents, as well as to identify the gaps and, thus, to provide recommendations for the research agenda. In particular, this scoping review aimed to develop a clear vision of which type of SR processes were targeted to enhance EXT symptoms during adolescence. We also examined which type of interventions were provided and for which type of population ranging from healthy adolescents (i.e., universal prevention), adolescents at risk or with light behavioral difficulties (i.e., selective prevention), offenders (i.e., indicated prevention), or adolescents presenting severe EXT symptoms (i.e., treatment).

## 2. Materials and Methods

### 2.1. Procedure

In accordance with our aim, we decided to conduct a scoping review [34,35,36]. The report follows almost all items of the PRISMA-ScR guidelines [37,38]. A scoping review may help to map the relevant literature available on a particular research area to identify key characteristics and gaps in the research area [39]. The methodology was based on the framework described by Arksey and O’Malley [34] and followed five key stages: (1) defining the research question, (2) identifying relevant studies, (3) selecting the studies, (4) extracting the data, and (5) summarizing and reporting the results. A scoping review has several strengths, such as being systematic, transparent, and replicable [35].

More specifically, studies were eligible for the present scoping review if they (1) included a sample of adolescents (mean age between 12 and 18); (2) described a non-pharmaceutical intervention on self-regulatory processes (i.e., cognitive, emotional, physiological, or social); (3) sample composed of adolescents suffering from externalizing disorders (e.g., CD, IED or ODD) or the intervention targeted EXT symptoms (e.g., aggression, conduct problems or offending behaviors). We imposed no restriction on the publication date or origin of studies. Studies focusing mainly on attention-deficit/hyperactivity disorder (ADHD), substance use, sexual offending, autism spectrum disorder, or intellectual disabilities were not taken into account as these are specific areas of research leading to abundant literature, which is beyond the scope of this review. We considered only peer-reviewed studies in English and French, however, with inclusive study designs ranging from case studies to randomized controlled trials.

All steps (e.g., study selection, data extraction) were conducted by at least two reviewers, independent from each other. Any differences between reviewers were discussed to achieve a consensus or were resolved by consulting a third reviewer.

### 2.2. Search Strategy

**Figure 1:**
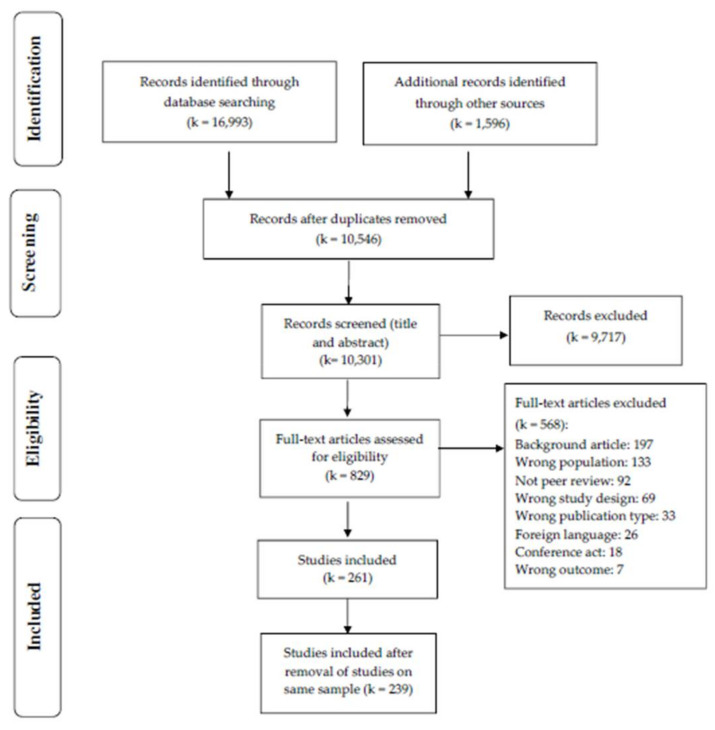
Flow diagram.

The systematic search was conducted, with the support of a medical librarian (JRA), on March 18, 2021, in the following bibliographic databases: Embase.com, Medline Ovid, PubMed (NOT medline[sb]), APA PsycINFO Ovid, Cochrane Library Wiley, Web of Science Core Collection, ProQuest Dissertations & Theses A&I and DART-Europe E-theses Portal. All searches were conducted without language or date restrictions. Additional records were identified through citation chaining based on the key references, using backward citation chasing (i.e., looking at the bibliography of the included studies) and forward citation chasing (i.e., reviewing references citing the included relevant articles). Forward citation chasing was performed in the Web of Science Core collection. The flow diagram is displayed in Figure 1. The full search strategies (including the algorithm of search as well as the database used) are available in Supplementary File S1.

First, the title and abstract of identified studies for possible inclusion (k = 10,546) were screened, which lead to 829 studies identified for full-text screening, from which we identified 261 studies that met inclusion criteria. Finally, we identified a number of studies (k = 22) that reported on the same sample. These studies were excluded to avoid artificial increased of their influence in the review. Therefore, we included 239 unique articles/studies (see Supplementary Table S1).

### 2.3. Study Categorization

For each article, we extracted information to describe the study. In particular, authors, publication year, country of origin, sample characteristics (e.g., age range, sample size), type of sample/stage of prevention or treatment (i.e., universal, selective, indicated prevention and treatment), study design (ranging from single case study to randomized control study). Finally, we categorized information about SR processes (i.e., cognitive, emotional, physiological, social), psychopathological symptoms (e.g., aggression, conduct problems, offending/delinquency, impulsivity), types of intervention (e.g., cognitive-based intervention, curriculum-based intervention, emotional-based intervention, mind-based intervention). Subcategories were developed through a top-down approach (i.e., use of the conceptual definition from the search algorithm) enriched by a bottom-up strategy through the screening of the included studies.

Below, we briefly defined the categories; for a detailed description, see Supplementary Table S2.

#### 2.3.1. Type of Samples

Each sample has been categorized according to the stage of prevention as follows: (a) universal prevention targets healthy adolescents or school-based interventions; (b) selective prevention focuses on adolescents who are at risk of starting a criminal career and/or with mild emotional, and behavioral difficulties; (c) indicated prevention is an intervention subsequent to the commission of an offense and concerns adolescents incarcerated in a juvenile facility or under probationary period; and (d) treatment, finally, concerns adolescent treated in child and adolescent mental health services for severe EXT symptoms or diagnoses such as CD, IED, and ODD.

#### 2.3.2. SR Processes

SR processes were categorized as follows: (1) cognition (e.g., executive functions, cognitive control); (2) emotion (e.g., emotion regulation, anger management); (3) social (e.g., peer or family support) and; (4) physiological (e.g., heart-rate variability).

#### 2.3.3. Psychopathological Symptoms

Psychopathological symptoms were categorized into EXT symptoms (e.g., conduct problems, aggressiveness, irritability/anger dysregulation, offending, impulsivity, substance use, attention-deficit/hyperactivity disorder (ADHD) symptoms, and sexual risky behaviors) and internalizing ones (i.e., self-injurious behaviors, anxiety, depressive symptoms, and somatization). In particular, regarding the main EXT symptoms, conduct problems characterized violation of social norms (e.g., disruptive behaviors in the classroom), whereas aggressiveness refers to hostility, intentional acts that verbally or physically harm others, as well as inappropriate interactions with others (e.g., conflict within the family). Irritability/anger dysregulation is linked to anger dysregulation and irritable mood (e.g., anger outburst). Offending is related to criminal activities or a violation of laws (e.g., violent crime, violation of societal norms). Impulsivity refers to acting without thinking (e.g., lack of premeditation, lack of anticipation of the consequences, risk-taking).

#### 2.3.4. Intervention Type

Interventions were categorized according to their main target, such as body, emotion, cognition, curriculum (i.e., multiple-stepped approach encompassing several interventions organized in time), family, social, mind (e.g., mindfulness, hypnosis), or externally mediated (e.g., animal-assisted therapy).

### 2.4. Data Synthesis

We provide descriptive statistics for the main information extracted from the studies and crossed some categories to allow for in-depth insights. Moreover, we conducted network analyses with the Igraph package [40] running on R v.3.6.0 [41] to illustrate the relationships between the interventions (type and stage) and psychopathological symptoms, which allow us to identify hubs and gaps.

## 3. Results

### 3.1. Descriptive

Our review includes 239 studies, mainly conducted in North America (see Table 1). Publications increased in number between the decades 1968–1980 and 2011–2020. The total number of participants in all studies was about 24,180 adolescents, from which about 13,341 received an intervention. In particular, studies conducted in North America involved 16,532 participants, those in Europe involved 4023 participants, those in Asia involved 1775 participants.

The participants were 12,446 males and 7663 females (NA = 4071). The age of participants ranged from 7 to 25 years, with a mean age of 14.6 (SD = 1.7). The majority of studies reported an intervention for both females and males (k = 128, 53.6%), whereas 72 studies were interventions for male-only (30.1%) and 10 studies for female-only (4.2%). A total of 29 studies (12.1%) lacked information about the sex of the participants. Randomized controlled trials and controlled trials represented almost 60% (k = 138) of the total study design. The majority of studies (about 70%, k = 162) concerned selective or indicated prevention stages.

Curriculum-based interventions were the most common type of intervention used in the studies. Cognitive-based interventions, mind-based interventions, and emotional-based interventions have increased drastically over the years (from 16.3%, 4.8%, and 0% in the 1968–1980s to 39.5%, 66.7%, and 46.1% in the 2010–2020s, respectively). Family-based interventions were more represented in the 2000 s while social-based interventions were declining (probably recently rather included in the curriculum-based interventions). Randomized controlled trials represented the main study design for almost every intervention ranging from 66.7% for family-based interventions, 45.5% for body-based interventions, 41.7% for externally mediated interventions and social-based interventions, 41.3% for curriculum-based interventions, 32.6% for cognitive-based interventions, 30.8% for emotional-based interventions and 23.8% for mind-based interventions (controlled trials represent 19% of these studies, whereas 33.3% had an open label).

Studies conducted in North America focused mainly on curriculum-based interventions (k = 50, 29.4%) and cognitive-based interventions (k = 31, 18.2%). European studies focused mainly on curriculum-based interventions (k = 14, 43.8%) and scarcely on emotional-based interventions (k = 1, 3.1%), family-based interventions (k = 1, 3.1%), and body-based interventions (k = 2, 6.3%), whereas no European studies focused on externally mediated interventions. Studies conducted in Asia mainly focused on emotional-based interventions (k = 5, 33.3%) and mind-based interventions (k = 4, 26.7%). In Australia, studies mainly focused on curriculum-based interventions (k = 7, 58.3%) and externally mediated interventions (k = 3, 25%).

Most studies focused on SR processes in the domain of cognition followed by emotion and social. Of note, physiological processes were scarcely taken into account in previous studies. Aggressiveness, conduct problems, and irritability/anger dysregulation were the most common EXT symptoms examined in previous studies. Although our scoping review did not target INT symptoms, anxiety and depressive symptoms have been measured in an important number of studies. Of notice is that INT symptoms have been examined mainly in association with aggressiveness and offending. Irritability/anger dysregulation and conduct problems in combination with INT symptoms have been examined in few studies.

### 3.2. Crossing Information and Network Analyses

#### 3.2.1. Intervention Stage by Origin

Regarding the association between the study’s origin and the stage of intervention, we observed that studies conducted in North America mainly targeted indicated prevention stage (71 studies, 41.8%) and selective prevention stage (54 studies, 31.8%), whereas only 16 studies (9.4%) targeted universal prevention stage. By contrast, studies conducted in Europe mainly targeted adolescents in treatment (10 studies, 31.3%), followed by universal prevention stage (8 studies, 25%) and indicated prevention stage (8 studies, 25%). Studies conducted in Asia, Australia, Africa, and South America targeted mainly the stage of selective prevention.

#### 3.2.2. Intervention Stage by Intervention Type

Specific types of intervention were more frequent in certain populations (see Table 2). Studies conducted in the universal prevention stage mainly focused on curriculum-based interventions and family-based interventions followed by mind-based interventions and social-based interventions. In the selective prevention stage, studies focused mainly on cognitive-based interventions, emotional-based interventions, and curriculum-based interventions. In the indicated prevention stage, studies mainly focused on curriculum-based interventions, followed by cognitive-based interventions, family-based interventions, and social-based interventions. Finally, the main intervention type in treatment studies was curriculum-based interventions and cognitive-based interventions.

#### 3.2.3. Intervention Type by Psychopathological Symptoms and SR Processes

Regarding the association between the type of intervention and SR processes (see Table 3), cognitive SR processes were mostly related to curriculum-based interventions and scarcely to body-based interventions or externally mediated based interventions. Social SR processes were included mainly in curriculum-based interventions, followed by family-based interventions and social-based interventions. However, emotional-based interventions, mind-based interventions, and body-based interventions were scarcely related to social SR processes. Emotional SR processes were mainly included in curriculum-based interventions. Physiological SR processes, which are examined in few studies, are mostly related to body-based interventions and were not taken into account in cognitive-based interventions, externally mediated interventions, family-based interventions, and social-based interventions.

**Figure 2:**
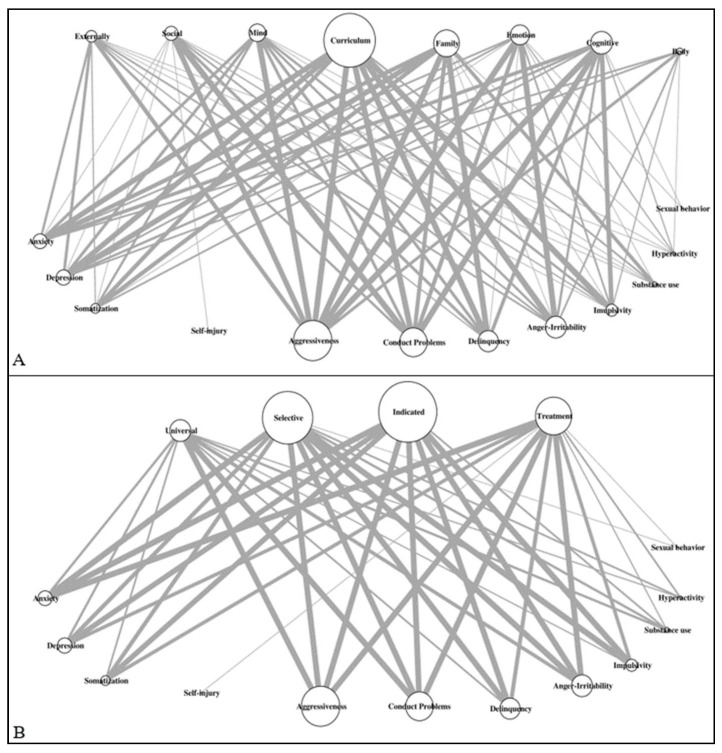
Illustration of the network between symptoms and type of intervention (**A**) or intervention stage; (**B**) Legend: upper part: type of intervention (**A**) or intervention stage (**B**); lower part: psychopathological symptoms. The size of the vertex is relative to the number of studies. The edge widths are relative to the number of studies (up to 10, for illustration purposes).

Regarding the association between psychopathological symptoms and the type of intervention, aggressiveness was the most studied symptom and was mostly represented in curriculum-based interventions. Conduct problems were mostly studied in curriculum-based interventions and were not taken into account in body-based interventions. Delinquency was mainly studied in curriculum-based interventions, followed by family-based interventions and scarcely in emotional-based interventions and externally mediated interventions. Impulsivity was mainly examined in curriculum-based interventions and cognitive-based interventions. Irritability/anger dysregulation was mostly related to curriculum-based interventions and emotional-based interventions and scarcely to family-based interventions. INT symptoms were mostly studied in curriculum-based interventions as well as family-based interventions and scarcely in social-based interventions (see Figure 2A).

#### 3.2.4. Intervention Stage by SR Processes and Psychopathological Symptoms

Regarding the association between SR processes and the stage of intervention, cognitive SR processes were mostly targeted in the indicated prevention stage (70 studies, 36.5%) and in the selective prevention stage (62 studies, 32.3%) and scarcely in the universal prevention stage (25 studies, 13%). Social SR processes were included mainly in the indicated prevention stage (40 studies, 40.1%) and scarcely in treatment (14 studies, 14.3%). Emotional SR processes were mostly represented in the indicated prevention stage (33 studies, 32%) and in the selective prevention stage (33 studies, 32%) and scarcely in the universal prevention stage (15 studies, 14.6%). Finally, physiological SR processes were included mainly in the indicated prevention stage (3 studies, 33.3%) and treatment (3 studies, 33.3%).

Regarding the association between the intervention stage and psychopathological symptoms, proportionally, aggressiveness was the most represented in the indicated prevention stage (34 studies, 32.4%) and at least in the universal prevention stage (19 studies, 18.1%). Conduct problems were mostly taken into account in the stage of selective (29 studies, 38.2%) and indicated (22 studies, 28.9%) prevention. Outcomes regarding delinquencies were mostly represented in the indicated prevention stage (34 studies, 61.8%). Impulsivity was examined in 13 studies (40.6%) from indicated prevention and in 10 studies of selective prevention (31.3%). Irritability/anger dysregulation was the most represented in selective (20 studies, 35.1%), indicated (17 studies, 29.8%), and treatment (13 studies, 22.8%). Internalizing symptoms (i.e., anxiety, depressive symptoms, somatization) were mostly present in indicated prevention stage, selective prevention stage as well as in treatment, but in a few studies of universal prevention. Figure 2B illustrates the network analyses resulting from the intervention stage crossed with psychopathological symptoms.

## 4. Discussion

This scoping review aims at mapping the non-pharmaceutical interventions targeting SR processes to alleviate EXT symptoms in adolescents, identifying critical research gaps and, thus, providing recommendations for the research agenda. We identified 239 studies, mainly from North America, highlighting an origin bias, including a total of 24,180 participants. Using descriptive statistics and network analyses, we concluded that the study design referred mainly to randomized controlled trials and controlled trials representing almost 60% (k = 138). The majority of studies concerned the population targeted by interventions in the stages of selective or indicated prevention (about 70%, k = 162), as well as curriculum-based interventions, were the most common type of intervention used in the studies. Cognitive-based interventions, mind-based interventions, and emotional-based interventions increased importantly over the years. The SR processes mostly targeted were cognitively measured, followed by emotional and social. Aggressiveness, conduct problems, and irritability/anger dysregulation were the most common EXT symptoms examined in previous studies. Although our scoping review did not target INT symptoms, anxiety and depressive symptoms have been measured in an important number of studies, as did manifest comorbidities. By contrast, our scoping review identified gaps by revealing both a scarcity of studies on the physiological SR processes, as well as on body-based interventions and externally mediated interventions. Moreover, tools such as biofeedback or neurofeedback and neuropsychological interventions (e.g., cognitive remediation) were not previously studied.

### 4.1. Hubs

Curriculum-based interventions were the most common type of intervention used in the studies. More specifically, such multiple-stepped interventions were mainly composed of cognitive-based interventions, emotional-based interventions, and social-based interventions. Thus, while social-based interventions have declined over the years, they now were included in curriculum-based interventions. Moreover, curriculum-based interventions included mainly anger control training or aggression replacement training, with cognitive training (e.g., problem-solving skills), social skills training, or relaxation program. Mind-based interventions had increased drastically from 1968 to 2020, underlying those interventions, such as mindfulness or yoga, become more popular. In the same way, externally mediated interventions were not considered as an intervention to enhance SR until the 2000s.

In terms of psychopathological symptoms, aggressiveness was the most EXT symptom examined in previous studies. This could be related to the fact that violence is the fourth leading cause of death among adolescents [42]. In this context, it is important to consider aggressiveness in a dimensional perspective (on a continuum from normal to pathological) and not in a categorical approach of EXT behaviors e.g., [18]. Moreover, our scoping review highlighted the frequent assessment of INT symptoms (i.e., anxiety and depressive symptoms as frequent comorbidities and that is in association with aggressiveness and offending). In the same line, Costello et al. [43] pointed out that adolescents suffering from EXT symptoms were also at high risk to present INT symptoms. This stressed the importance of adopting a broad perspective when helping adolescents.

### 4.2. Origin Bias and Cultural Aspects

In our scoping review, a substantial number of studies were conducted in North America (k = 170), highlighting the unequal production of knowledge from certain countries and cultures. Indeed, the interest of most journal remain American-centric, and North America is overrepresented in terms of the number of articles produced [44]. Moreover, scientific productivity has long characterized research in North America [45,46]. This might be explained by the fact that research has been much better funded. People are more interested in participating in research projects. As well, medicine, and especially psychiatry, is to a higher degree “evidence-based”.

We also observed some differences across the European Union. Indeed, in Europe, studies were conducted mainly in the Netherlands, Spain, and United Kingdom. Interestingly, in Western Europe, no studies have been conducted in France and only one single study in Germany.

Moreover, we observed that in North America, studies focused mainly on the selective and indicated prevention stage, whereas studies in Europe examined mainly treatment. This result might come from the cultural and sociological way to understand EXT symptoms ranging from psychiatric to criminal perspectives. Indeed, legal traditions and forensic psychiatric systems differ, as do the legal response toward offenders: specialized forensic mental health care or imprisonment. Countries adopting an Anglo-Saxon law tradition, such as the United Kingdom, Ireland, United States, Canada, Australia, or South Africa, were observed to less consider the effect of mental disorder on the criminal responsibility of offenders in the trial [47]. Furthermore, while European forensic psychiatrists focus on the treatment of mentally disordered offenders, the role of the forensic psychiatrist is slightly different in the United States tradition as it is more embedded in the legal context [47]. Therefore, studies conducted in these countries rarely targeted adolescents in treatment (but more in indicated prevention) and revealed the influence of the law traditions on practices with regard to criminality proceedings [47].

### 4.3. Gaps and Recommendation of Research Agenda

Neuropsychological (e.g., cognitive remediation), as well as neurofeedback interventions, were scarcely studied (only one pilot study on cognitive remediation) [48]. Regarding the enhanced brain plasticity observed in adolescence [31] as well as the deficits in executive functions in individuals with antisocial behaviors e.g., [49,50], this may be an important area of research in the future. In adults, neuropsychological interventions e.g., [51], have shown great benefits but were scarcely studied [52].

The majority of studies concerned the stages of selective or indicated prevention, and not many in the universal prevention stage. However, a converging body of research suggests that risk factors leading to a long-term criminal career can be identified during childhood with reasonable accuracy e.g., [53]. Hence the importance of promoting an effective prevention program (i.e., universal prevention stage) to intervene in the school years and improving self-regulatory processes in order to alleviate EXT symptoms in adolescents.

Moreover, it seems that the percentage of RCT is lower in mind-based interventions, body-based interventions, and externally mediated interventions, pleading for future studies with a more rigorous design. Physiological SR processes should be more deeply studied and in particular in studies examining intervention based on cognition, externally mediated, family, and social. Moreover, cognitive SR processes should be more extensively studied in interventions based on body or externally mediated. As well, social SR processes should be studied in interventions based on mind, emotion and body, to allow individuals to adapt their thoughts and their behaviors to the ever-changing environment. Adopting the perspective of the polyvagal theory e.g., [22], may help to develop such studies within a more integrative theoretical perspective, including psychological (i.e., cognitive and emotional), physiological, and social SR processes.

In the future, systematic reviews and meta-analyses should help to harmonize the practice by assessing the effectiveness and the moderating effects of the interventions, as well as evaluating the biases systematically. Such reviews will provide important insights to develop guidelines to help these adolescents.

### 4.4. Comparison with Previous Reviews and Meta-Analyses

Compared with previous reviews taking into account only the psychological or social processes of SR [23,54], the current scoping review adopted a broader perspective on SR processes, including also physiological markers. In particular, previous meta-analyses observed that during childhood or adolescence, self-control training improves self-control skills [55,56,57], which also decreases delinquency [23]. Likewise, a meta-analysis [54] on interventions targeting SR skills in children and adolescents reported that SR interventions are effective in enhancing SR skills as well as on reducing or preventing EXT symptoms e.g., [58,59]. Of note, Pandey and colleagues [54] have also highlighted the variety of interventions and reported that curriculum-based interventions were the most common type of intervention used. Nevertheless, a previous meta-analysis [60] stated that interventions preventing EXT symptoms are rather meager, which is partially due to insufficient evidence regarding the long-term benefits.

Moreover, to the best of our knowledge, no previous reviews clearly separate the stage of interventions to examine that type of interventions were provided to which stage of intervention. We also included all types of intervention and did not limit our review to randomized controlled trials. This scoping review, thus, evokes a larger view (including 239 studies instead of 50 in the broader previous meta-analysis) and allows, through the network analyses, to map the existing possible interventions allowing to enhance SR to reduce EXT symptoms in the context of adolescence.

### 4.5. Limitations

This scoping review has been designed to map the interventions on SR to reduce EXT symptoms and to identify the gaps but did not assess the effectiveness of the intervention, which should be investigated in future systematic reviews or meta-analyses. This should help to harmonize the practice by assessing the effectiveness and moderating effects of the intervention. Such systematic reviews and/or meta-analyses will provide important insight to develop guidelines to help such adolescents. Moreover, we limited our scoping review to adolescents; thus, further reviews should focus on the children population or adults. Our scoping review was also limited to published literature in the English and French languages. We may have therefore missed some information published in other languages; however, we did not think that it taints the global observed picture. As we excluded studies focusing mainly on ADHD, substance use, sexual offending, autism spectrum disorder, or intellectual disabilities, we might have observed a truncated picture. However, EXT behaviors in these disorders are specific and out of the scope of this review.

## 5. Conclusions

This scoping review aimed to map non-pharmaceutical interventions targeting self-regulatory processes to improve EXT symptoms in adolescents. Our scoping review stressed the heterogeneity and plethora of interventions with the objective to enhance SR skills. Curriculum-based interventions (i.e., multiple approaches integrating various interventions organized in time) appear to be the most common type of non-pharmaceutical interventions proposed to reduce EXT symptoms, in particular aggressiveness, conduct problems, and delinquency. Our review focused on EXT symptoms but revealed that an important number of studies also measured INT symptoms as comorbidities. This review also identified several gaps in the literature and highlighted a scarcity of studies examining physiological processes, as well as on body-based interventions and externally mediated interventions. In particular, we concluded a lack concerning some tools, such as biofeedback or neurofeedback techniques, as well as neuropsychological interventions. Moreover, further studies in mind-based interventions, body-based interventions, and externally mediated interventions should adopt more rigorous designs to provide more reliable evidence. This scoping review thus allowed identifying important areas of future research.

## Figures and Tables

**Table 1:** Descriptive.

Variables	Categories	*n* (%) of Studies
Publication’s year	1968–1980	24 (10.0)
	1981–1990	32 (13.4)
	1991–2000	39 (16.3)
	2001–2010	48 (20.1)
	2011–2020	96 (40.1)
Study’s origin	North America	170 (71.1)
	Europe	32 (13.4)
	Asia	15 (6.3)
	Australia	12 (5.0)
	Africa	3 (1.3)
	South America	1 (0.4)
	Not reported	6 (2.5)
Study design	Randomized controlled trial	96 (40.2)
	Open label	50 (20.9)
	Controlled trial	42 (17.6)
	ABAB design	17 (7.1)
	Single case	11 (4.6)
	Multiple cases	9 (3.8)
	Qualitative	5 (2.1)
	ABBA crossover	3 (1.3)
	Others	6 (2.5)
Intervention stage	Universal prevention	30 (12.6)
	Selective prevention	75 (31.4)
	Indicated prevention	87 (36.4)
	Treatment	47 (19.7)
Intervention type	Curriculum-based interventions	75 (31.4)
	Cognitive-based interventions	43 (18.0)
	Family-based interventions	27 (11.3)
	Emotional-based interventions	26 (10.9)
	Social-based interventions	24 (10.0)
	Mind-based interventions	21 (8.8)
	Externally mediated interventions	12 (5.0)
	Body-based interventions	11 (4.6)
SR process *	Cognition	192 (47.7)
	Emotion	103 (25.6)
	Social	98 (24.4)
	Physiological	9 (2.2)
EXT symptom *	Aggressiveness	105 (29.8)
	Conduct problems	76 (21.6)
	Irritability/anger dysregulation	57 (16.2)
	Delinquency/offending	55 (15.6)
	Impulsivity	32 (9.1)
	Substance use	16 (4.5)
	Hyperactivity	9 (2.6)
	Sexual risky behaviors	2 (0.6)
INT symptom *	Depressive symptoms	39 (37.8)
	Anxiety	38 (36.9)
	Somatization	25 (24.3)
	Self-injurious behaviors	1 (1.0)

* Studies may include more than one process/symptom. SR: self-regulation; EXT: externalizing; INT: internalizing.

**Table 2:** Number/percentage of studies classified by the type of intervention and its stage.

		Type of Intervention								
		Body	Cognitive	Curriculum	Emotional	Externally Mediated	Family	Mind	Social	Total
Stage	Universal	1/0.4	1/0.4	9/3.8	2/0.8	0/0	8/3.3	5/2.1	4/1.7	30/12.6
	Selective	2/0.8	17/7.1	24/10.0	14/5.9	4/1.7	5/2.1	4/1.7	5/2.1	75/31.4
	Indicated	6/2.5	14/5.9	28/11.7	5/2.1	5/2.1	11/4.6	7/2.9	11/4.6	87/36.4
	Treatment	2/0.8	11/4.6	14/5.9	5/2.1	3/1.3	3/1.3	5/2.1	4/1.7	47/19.7
	Total	11/4.5	43/18	75/31.4	26/10.9	12/5.1	27/11.3	21/8.8	24/10.1	239/100

Data expressed in *n*/%. The number of studies was transformed into a percentage in order to appreciate the repartition of the studies, where 100% refer to the 239 studies.

**Table 3:** Number/percentage of occurrence of SR processes and psychopathological symptoms in each type of intervention.

		Intervention Type								
		Body	Cognitive	Curriculum	Emotional	Externally Mediated	Family	Mind	Social	Total
Self-regulation	Cognition	7/1.7	42/10.4	68/17.0	14/3.5	6/1.5	17/4.2	19/4.7	19/4.7	192/47.8
	Social	2/0.5	8/2.0	33/8.2	5/1.2	7/1.7	21/5.2	4/1.0	18/4.5	98/24.4
	Emotion	5/1.2	9/2.2	36/9.0	23/5.7	9/2.2	9/2.2	11/2.7	1/0.2	103/25.6
	Physiological	5/1.2	0/0	2/0.5	1/0.2	0/0	0/0	1/0.2	0/0	9/2.2
	Total	19/4.7	59/14.7	139/34.6	43/10.7	22/5.5	47/11.	35/8.7	38/9.4	402/100
Symptoms	Aggressiveness	5/1.1	12/2.6	34/7.5	12/2.6	7/1.5	14/3.1	12/2.6	9/2.0	105/23.2
	Conduct problems	0/0	12/2.6	27/6.0	7/1.5	4/0.9	8/1.8	6/1.3	12/2.6	76/16.8
	Delinquency	0/0	6/1.3	19/4.2	1/0.2	2/0.4	16/3.5	5/1.1	5/1.1	54/11.9
	Hyperactivity	1/0.2	2/0.4	0/0	2/0.4	1/0.2	1/0.2	2/0.4	0/0	9/2.0
	Impulsivity	2/0.4	8/1.8	11/2.4	3/0.7	1/0.2	1/0.2	4/0.9	2/0.4	32/7.1
	Irritability/anger dys.	3/0.7	4/0.9	21/4.6	17/3.8	3/0.7	1/0.2	4/0.9	3/0.7	56/12.4
	Substance use	0/0	0/0	4/0.9	1/0.2	1/0.2	8/1.8	1/0.2	1/0.2	16/3.5
	Sexual risky behaviors	0/0	1/0.2	0/0	1/0.2	0/0	0/0	0/0	0/0	2/0.4
	Self-injurious behaviors	0/0	0/0	0/0	0/0	0/0	0/0	0/0	1/0.2	1/0.2
	Anxiety	3/0.7	6/1.3	9/2.0	3/0.7	4/0.9	7/1.5	5/1.1	1/0.2	38/8.4
	Depressive symptoms	3/0.7	4/0.9	9/2.0	3/0.7	5/1.1	9/2.0	5/1.1	1/0.2	39/8.6
	Somatization	0/0	3/0.7	7/1.5	1/0.2	2/0.4	7/1.5	4/0.9	1/0.2	25/5.5
	Total	17/3.8	58/12.8	141/31.1	51/11.3	30/6.6	72/15.9	48/10.6	36/7.9	453/100

Data expressed in *n*/%. The number of occurrence of SR processes (100% referring to 402 occurrences) or psychopathological (100% corresponding to 453 occurrences) were transformed into percentages in order to appreciate their repartition in each type of intervention.

## Data Availability

Not applicable.

## Supplementary Materials

The following are available online at https://www.mdpi.com/article/10.3390/biomedicines9091081/s1, File S1: Bibliographic database search strategies. Table S1: Included studies (*n* = 239). Table S2: Definition of the categories.

## References

[1] Agnew R. (2003). An integrated theory of the adolescent peak in offending. Youth Soc..

[2] Cohen M.A., Piquero A.R. (2008). New evidence on the monetary value of saving a high risk youth. J. Quant. Criminol..

[3] Storz R. (2007). Evolution de la Délinquance Juvénile: Jugements Pénaux Des Adolescents, de 1946 à 2004.

[4] Campbell S.B. (1995). Behavior problems in preschool children: A review of recent research. J. Child Psychol. Psychiatry.

[5] Fairchild G., Passamonti L., Hurford G., Hagan C., von dem Hagen E., Van Goozen S.H., Goodyer I.M., Calder A.J. (2011). Brain structure abnormalities in early-onset and adolescent-onset conduct disorder. Am. J. Psychiatry.

[6] Frick P.J., Viding E. (2009). Antisocial behavior from a developmental psychopathology perspective. Dev. Psychopathol..

[7] Frick P.J., White S.F. (2008). Research Review: The importance of callous-unemotional traits for developmental models of aggressive and antisocial behavior. J. Child Psychol. Psychiatry.

[8] Moffitt T.E., Lahey B.B., Moffitt T.E., Caspi A. (2003). Life-course persistent and adolescence-limited antisocial behavior: A 10-year research review and research agenda. Causes of Conduct Disorder and Juvenile Delinquency.

[9] Moffitt T.E., Cicchetti D., Cohen J. (2006). Life-course persistent versus adolescence-limited antisocial behavior. Developemental Psychaphtology, Risk, Disorder, and Adaptation.

[10] Pardini D.A., Frick P.J. (2013). Multiple developmental pathways to conduct disorder: Current conceptualizations and clinical implications. J. Can. Acad. Child Adolesc. Psychiatry.

[11] Gottfredson M.R., Hirschi T. (1990). A General Theory of Crime.

[12] Heatherton T.F., Wagner D.D. (2011). Cognitive neuroscience of self-regulation failure. Trends Cogn. Sci..

[13] Perry N.B., Calkins S.D., Dollar J.M., Keane S.P., Shanahan L. (2017). Self-regulation as a predictor of patterns of change in externalizing behaviors from infancy to adolescence. Dev. Psychopathol..

[14] Vazsonyi A.T., Mikuška J., Kelley E.L. (2017). It’s time: A meta-analysis on the self-control-deviance link. J. Crim. Justice.

[15] Odgers C.L., Moffitt T.E., Broadbent J.M., Dickson N., Hancox R.J., Harrington H., Poulton R., Sears M.R., Thomson W.M., Caspi A. (2008). Female and male antisocial trajectories: From childhood origins to adult outcomes. Dev. Psychopathol..

[16] Frick P.J., Thornton L.C., Centifanti L.C., Williams D.M. (2017). A Brief history of the diagnostic classification of childhood externalizing disorders. The Wiley Handbook of Developmental Psychopathology.

[17] American Psychiatric Association (2013). Diagnostic and Statistical Manual of Mental Disorders.

[18] Kotov R., Krueger R.F., Watson D., Achenbach T.M., Althoff R.R., Bagby R.M., Brown T.A., Carpenter W.T., Caspi A., Clark L.A. (2017). The Hierarchical Taxonomy of Psychopathology (HiTOP): A dimensional alternative to traditional nosologies. J. Abnorm. Psychol..

[19] Insel T.R. (2014). The NIMH research domain criteria (RDoC) project: Precision medicine for psychiatry. Am. J. Psychiatry.

[20] Coccaro E.F. (2012). Intermittent explosive disorder as a disorder of impulsive aggression for DSM-5. Am. J. Psychiatry.

[21] Nigg J.T. (2016). Annual Research Review: On the relations among self-regulation, self-control, executive functioning, effortful control, cognitive control, impulsivity, risk-taking, and inhibition for developmental psychopathology. J. Child Psychol. Psychiatry.

[22] Porges S.W. (2007). The polyvagal perspective. Biol. Psychol..

[23] Piquero A.R., Jennings W.G., Farrington D.P., Diamond B., Gonzalez J.M.R. (2016). A meta-analysis update on the effectiveness of early self-control improvement programs to improve self-control and reduce delinquency. J. Exp. Criminol..

[24] Beauchaine T.P., Gatzke-Kopp L., Mead H.K. (2007). Polyvagal Theory and developmental psychopathology: Emotion dysregulation and conduct problems from preschool to adolescence. Biol. Psychol..

[25] Beauchaine T.P., Neuhaus E., Brenner S.L., Gatzke-Kopp L. (2008). Ten good reasons to consider biological processes in prevention and intervention research. Dev. Psychopathol..

[26] Habersaat S., Boonmann C., Schmeck K., Stéphan P., Francescotti E., Fegert J.M., Perler C., Gasser J., Schmid M., Urben S. (2018). Differences and similarities in predictors of externalizing behavior problems between boys and girls: A 1-year follow-up study. Crim. Justice Behav..

[27] Urben S., Stéphan P., Habersaat S., Francescotti E., Fegert J.M., Schmeck K., Perler C., Gasser J., Schmid M. (2016). Examination of the importance of age of onset, callous-unemotional traits and anger dysregulation in youths with antisocial behaviors. Eur. Child Adolesc. Psychiatry.

[28] Mantzouranis G., Zimmermann G. (2010). Prendre des risques, ça rapporte? Conduites à risques et perception des risques chez des adolescents tout-venant. Neuropsychiatr. De L’enfance Et De L’adolescence.

[29] Steinberg L. (2008). A social neuroscience perspective on adolescent risk-taking. Dev. Rev..

[30] Somerville L.H., Jones R.M., Casey B.J. (2010). A time of change: Behavioral and neural correlates of adolescent sensitivity to appetitive and aversive environmental cues. Brain Cogn..

[31] Fuhrmann D., Knoll L.J., Blakemore S.-J. (2015). Adolescence as a sensitive period of brain development. Trends Cogn. Sci..

[32] Correll C.U., Cortese S., Croatto G., Monaco F., Krinitski D., Arrondo G., Ostinelli E.G., Zangani C., Fornaro M., Estradé A. (2021). Efficacy and acceptability of pharmacological, psychosocial, and brain stimulation interventions in children and adolescents with mental disorders: An umbrella review. World Psychiatry.

[33] Ipser J., Stein D.J. (2006). Systematic review of pharmacotherapy of disruptive behavior disorders in children and adolescents. Psychopharmacology.

[34] Arksey H., O’Malley L. (2005). Scoping studies: Towards a methodological framework. Int. J. Soc. Res. Methodol..

[35] Grant M.J., Booth A. (2009). A typology of reviews: An analysis of 14 review types and associated methodologies. Heal. Inf. Libr. J..

[36] Munn Z., Stern C., Aromataris E., Lockwood C., Jordan Z. (2018). What kind of systematic review should I conduct? A proposed typology and guidance for systematic reviewers in the medical and health sciences. BMC Med Res. Methodol..

[37] Moher D., Liberati A., Tetzlaff J., Altman D.G. (2009). Preferred reporting items for systematic reviews and meta-analyses: The PRISMA statement. BMJ.

[38] Tricco A.C., Lillie E., Zarin W., O’Brien K.K., Colquhoun H., Levac D., Moher D., Peters M.D.J., Horsley T., Weeks L. (2018). PRISMA extension for scoping reviews (PRISMA-ScR): Checklist and explanation. Ann. Intern. Med..

[39] Daudt H.M.L., Van Mossel C., Scott S.J. (2013). Enhancing the scoping study methodology: A large, inter-professional team’s experience with Arksey and O’Malley’s framework. BMC Med Res. Methodol..

[40] Csardi G., Nepusz T. (2006). The igraph software package for complex network research. Int. J. Complex Syst..

[41] Team R Core (2018). R. A Language and Environment for Statistical Computing.

[42] World Health Organization (2005). Politiques et Plans Relatifs à la Santé Mentale de L’Enfant et de L’Adolescent.

[43] Costello E.J., Mustillo S., Erkanli A., Keeler G., Angold A. (2003). Prevalence and development of psychiatric disorders in childhood and adolescence. Arch. Gen. Psychiatry.

[44] Wai-Chung Y.H. (2001). Redressing the geographical bias in social science knowledge. Environ. Plan. A Econ. Space.

[45] Fanelli D., Costas R., Ioannidis J.P.A. (2017). Meta-assessment of bias in science. Proc. Natl. Acad. Sci. USA.

[46] Fanelli D., Ioannidis J.P.A. (2013). US studies may overestimate effect sizes in softer research. Proc. Natl. Acad. Sci. USA.

[47] Völlm B.A., Clarke M., Herrando V.T., Seppänen A.O., Gosek P., Heitzman J., Bulten E. (2017). European Psychiatric Association (EPA) guidance on forensic psychiatry: Evidence based assessment and treatment of mentally disordered offenders. Eur. Psychiatry.

[48] Rowlands A., Fisher M., Mishra J., Nahum M., Brandrett B., Reinke M., Caldwell M., Kiehl K.A., Vinogradov S. (2020). Cognitive training for very high risk incarcerated adolescent males. Front. Psychiatry.

[49] Morgan A.B., Lilienfeld S.O. (2000). A meta-analytic review of the relation between antisocial behavior and neuropsychological measures of executive function. Clin. Psychol. Rev..

[50] Ogilvie J.M., Stewart A.L., Chan R.C.K., Shum D.H.K. (2011). Neuropsychological measures of executive function and antisocial behavior: A meta-analysis*. Criminology.

[51] Baskin-Sommers A.R., Curtin J.J., Newman J.P. (2015). Altering the cognitive-affective dysfunctions of psychopathic and externalizing offender subtypes with cognitive remediation. Clin. Psychol. Sci..

[52] Bootsman F. (2019). Neurobiological intervention and prediction of treatment outcome in the juvenile criminal justice system. J. Crim. Justice.

[53] Haapasalo J., Tremblay R.E. (1994). Physically aggressive boys from ages 6 to 12: Family background, parenting behavior, and prediction of delinquency. J. Consult. Clin. Psychol..

[54] Pandey A., Hale D., Das S., Goddings A.-L., Blakemore S.-J., Viner R.M. (2018). Effectiveness of universal self-regulation–based interventions in children and adolescents. A systematic review and meta-analysis. JAMA Pediatr..

[55] Friese M., Frankenbach J., Job V., Loschelder D.D. (2017). Does self-control training improve self-control? A meta-analysis. Perspect. Psychol. Sci..

[56] Hagger M.S., Wood C., Stiff C., Chatzisarantis N.L.D. (2010). Ego depletion and the strength model of self-control: A meta-analysis. Psychol. Bull..

[57] Inzlicht M., Berkman E. (2015). Six questions for the resource model of control (and some answers). Soc. Pers. Psychol. Compass.

[58] Mason W.A., Fleming C.B., Ringle J.L., Thompson R.W., Haggerty K.P., Snyder J.J. (2014). Reducing risks for problem behaviors during the high school transition: Proximal outcomes in the common sense parenting trial. J. Child Fam. Stud..

[59] Webster-Stratton C., Reid M.J., Stoolmiller M. (2008). Preventing conduct problems and improving school readiness: Evaluation of the Incredible Years Teacher and Child Training Programs in high-risk schools. J. Child Psychol. Psychiatry.

[60] Smedler A.-C., Hjern A., Wiklund S., Anttila S., Pettersson A. (2014). Programs for prevention of externalizing problems in children: Limited evidence for effect beyond 6 months post intervention. Child Youth Care Forum.

