# Supplementary material for: Non-Pharmaceutical Interventions for Self-Regulatory Failures in Adolescents Suffering from Externalizing Symptoms: A Scoping Review
Source: Biomedicines. 2021 Aug 24;9(9):1081. doi: 10.3390/biomedicines9091081 (PMC8466021; doi:10.3390/biomedicines9091081)
Supplement: Supplementary file 1 [file biomedicines-09-01081-s001.zip › Supplementary_file_1.pdf]

Supplementary file 1: Non-pharmaceutical interventions for self-regulatory failures in adolescents suffering from externalizing symptoms: A scoping review

Bibliographic database search strategies

Note: the research strategies were peer reviewed by another information specialist prior to execution using the PRESS Checklist : McGowan J, Sampson M, Salzwedel DM, Cogo E, Foerster V, Lefebvre C. (2016). PRESS Peer Review of Electronic Search Strategies: 2015 Guideline Statement. *Journal of Clinical Epidemiology*, *75*, 40-46.

Embase.com

Last Content Update, 5 Oct 2020 01:31:40 GMT
4311 references found

('oppositional defiant disorder'/de OR 'intermittent explosive disorder'/de OR 'conduct disorder'/de OR 'antisocial personality disorder'/de OR 'behavior disorder'/de OR 'impulse control disorder'/exp OR 'disruptive behavior'/exp OR 'impulsiveness'/de OR 'inhibition (psychology)'/exp OR 'sensation seeking'/de OR 'high risk behavior'/de OR 'irritability'/de OR 'aggression'/exp OR 'theft'/de OR 'antisocial behavior'/exp OR 'offender'/de OR (externalizing OR externalising OR ODD OR ((oppositional OR defiant) NEXT/1 disorder*) OR "intermittent explosive disorder" OR "conduct disorder*" OR ((sociopath* OR psychopath* OR antisocial OR dyssocial) NEXT/1 (behav* OR personalit*)) OR sociopathy OR ((aberrant OR deviant OR disturb*) NEXT/1 behav*) OR (behav* NEXT/1 (aberration OR disorder* OR disturbance OR crisis)) OR runaway OR elopement OR "impulse control disorder*" OR "conduct disorder*" OR ((disruptive OR problem* OR dysfunctional) NEAR/3 behav*) OR problembehav* OR impulsiv* OR impulsigenic OR disinhibition OR inhibition OR "sensation seek*" OR risk-taking OR "risk behav*" OR "risky behav*" OR irritability OR "irritable mood*" OR ((defiant OR hostile) NEXT/1 behav*) OR aggress* OR counteraggress* OR anger OR "angry outbursts" OR provocation* OR deceitful* OR theft* OR stealing* OR ((antisocial OR anti-social OR asocial* OR deviant) NEAR/3 (behav* OR conduct* OR personalit* OR reaction*)) OR "social behav* disorder*" OR misbehav* OR misconduct* OR delinquency OR delinquent* OR offender* OR callous-unemotional OR "callous and unemotional"):ab,ti,kw) AND ('self control'/exp OR 'executive function'/de OR 'metacognition'/exp OR 'ego'/de OR 'autoregulation'/exp OR 'heart rate'/de OR 'heart rate variability'/de OR 'respiratory sinus arrhythmia'/de OR 'vagus tone'/de OR 'pressoreceptor reflex'/de OR 'autonomic nervous system'/de OR 'adrenergic system'/de OR (self-control OR "self manag*" OR "executive function*" OR "cognitive control" OR ((executive OR proactive OR reactive) NEXT/1 control) OR "effortful control" OR metacognition OR metacognitive OR meta-cognit* OR ((emotion* OR affect) NEXT/1 regulation) OR "emotional control" OR (control NEXT/3 emotion*) OR "ego control" OR "ego undercontrol" OR "ego resiliency" OR "ego depletion" OR autoregulation OR self-regulat* OR homeostasis OR ((cardiac OR heart) NEXT/1 (frequenc* OR rate*)) OR "respiratory sinus arrhythmia" OR "vagus tone" OR "vagal tone" OR vagotonus OR "vagus nerve tone" OR baroreflex* OR "pressoreceptor reflex" OR "baroceptor reflex" OR "baroreceptor reflex" OR "pressor reflex" OR "pressure reflex" OR "baroreceptor reflex" OR ((autonom* OR vegetative) NEXT/3 system) OR ((adrenergic OR

sympath* OR orthosympath*) NEXT/3 (mechanism OR system))):ab,ti,kw) AND ('juvenile'/de OR 'adolescent'/exp OR 'young adult'/de OR 'adolescence'/exp OR 'child psychiatry'/de OR 'juvenile delinquency'/de OR (adolescent* OR adolescence OR preadolescen* OR pre-adolescen* OR teen* OR juvenile OR youth* OR "young adult*"):ab,ti,kw) AND ('psychotherapy'/exp OR 'physical medicine'/exp OR 'alternative medicine'/exp OR 'biofeedback'/exp OR 'respiration control'/de OR (monitor* OR manag* OR psychotherap* OR therap* OR intervention* OR treatment* OR training* OR exercise* OR technique* OR respiration* OR breathing OR rehabilitation* OR approach* OR tool* OR remediation* OR healing OR biofeedback OR "bio feed back" OR bio-feedback OR biofeed-back):ab,ti,kw) NOT ([animals]/lim NOT [humans]/lim)

Medline Ovid SP

Ovid MEDLINE(R) and Epub Ahead of Print, In-Process & Other Non-Indexed Citations and Daily 1946 to October 01, 2020
3668 references found

("Attention Deficit and Disruptive Behavior Disorders"/ OR "Conduct Disorder"/ OR "Disruptive, Impulse Control, and Conduct Disorders"/ OR "Antisocial Personality Disorder"/ OR "Problem Behavior"/ OR "Impulsive Behavior"/ OR exp "Inhibition, Psychological"/ OR "Irritable Mood"/ OR exp "Aggression"/ OR "Theft"/ OR "Social Behavior Disorders"/ OR "Juvenile Delinquency"/ OR "Child Behavior Disorders"/ OR "Risk-Taking"/ OR (externalizing OR externalising OR ODD OR ((oppositional OR defiant) ADJ1 disorder*) OR "intermittent explosive disorder" OR "conduct disorder*" OR ((sociopath* OR psychopath* OR antisocial OR dyssocial) ADJ1 (behav* OR personalit*)) OR sociopathy OR ((aberrant OR deviant OR disturb*) ADJ1 behav*) OR (behav* ADJ1 (aberration OR disorder* OR disturbance OR crisis)) OR runaway OR elopement OR "impulse control disorder*" OR "conduct disorder*" OR ((disruptive OR problem* OR dysfunctional) ADJ3 behav*) OR problembehav* OR impulsiv* OR impulsigenic OR disinhibition OR inhibition OR "sensation seek*" OR risk-taking OR "risk behav*" OR "risky behav*" OR irritability OR "irritable mood*" OR ((defiant OR hostile) ADJ1 behav*) OR aggress* OR counteraggress* OR anger OR "angry outbursts" OR provocation* OR deceitful* OR theft* OR stealing* OR ((antisocial OR anti-social OR asocial* OR deviant) ADJ3 (behav* OR conduct* OR personalit* OR reaction*)) OR "social behav* disorder*" OR misbehav* OR misconduct* OR delinquency OR delinquent* OR offender* OR callous-unemotional OR "callous and unemotional").ab,ti,kf.) AND (exp "Self-Control"/ OR "Executive Function"/ OR "Metacognition"/ OR exp "Ego"/ OR "Homeostasis"/ OR "Baroreflex"/ OR exp "Heart Rate"/ OR "Autonomic Nervous System"/ OR (self-control OR "self manag*" OR "executive function*" OR "cognitive control" OR ((executive OR proactive OR reactive) ADJ1 control) OR "effortful control" OR metacognition OR metacognitive OR meta-cognit* OR ((emotion* OR affect) ADJ1 regulation) OR "emotional control" OR (control ADJ3 emotion*) OR "ego control" OR "ego undercontrol" OR "ego resiliency" OR "ego depletion" OR autoregulation

OR self-regulat* OR homeostasis OR ((cardiac OR heart) ADJ1 (frequenc* OR rate*)) OR "respiratory sinus arrhythmia" OR "vagus tone" OR "vagal tone" OR vagotonus OR "vagus nerve tone" OR baroreflex* OR "pressoreceptor reflex" OR "baroceptor reflex" OR "baroreceptor reflex" OR "pressor reflex" OR "pressure reflex" OR "baroreceptor reflex" OR ((autonom* OR vegetative) ADJ3 system) OR ((adrenergic OR sympath* OR orthosympath*) ADJ3 (mechanism OR system))).ab,ti,kf.) AND ("Adolescent"/ OR "Young Adult"/ OR "Adolescent Psychiatry"/ OR "Psychology, Adolescent"/ OR "Juvenile Delinquency"/ OR (adolescent* OR adolescence OR preadolescen* OR pre-adolescen* OR teen* OR juvenile OR youth* OR "young adult*").ab,ti,kf.) AND (exp "Psychotherapy"/ OR exp "Physical and Rehabilitation Medicine"/ OR exp "Complementary Therapies"/ OR (monitor* OR manag* OR psychotherap* OR therap* OR intervention* OR treatment* OR training* OR exercise* OR technique* OR respiration* OR breathing OR rehabilitation* OR approach* OR tool* OR remediation* OR healing OR biofeedback OR "bio feed back" OR bio-feedback OR biofeed-back).ab,ti,kf.) NOT (exp animals/ NOT humans.sh.)

PubMed (NOT medline[sb])

October 5, 2020
651 references found

(externalizing[tiab] OR externalising[tiab] OR ODD[tiab] OR ((oppositional[tiab] OR defiant[tiab]) AND disorder*[tiab]) OR "intermittent explosive disorder"[tiab] OR conduct disorder*[tiab] OR ((sociopath*[tiab] OR psychopath*[tiab] OR antisocial[tiab] OR dyssocial[tiab]) AND (behav*[tiab] OR personalit*[tiab])) OR sociopathy[tiab] OR ((aberrant[tiab] OR deviant[tiab] OR disturb*[tiab]) AND behav*[tiab]) OR (behav*[tiab] AND (aberration[tiab] OR disorder*[tiab] OR disturbance[tiab] OR crisis[tiab])) OR runaway[tiab] OR elopement[tiab] OR impulse control disorder*[tiab] OR conduct disorder*[tiab] OR ((disruptive[tiab] OR problem*[tiab] OR dysfunctional[tiab]) AND behav*[tiab]) OR impulsiv*[tiab] OR impulsigenic[tiab] OR disinhibition[tiab] OR inhibition[tiab] OR sensation seek*[tiab] OR risk-taking[tiab] OR risk behav*[tiab] OR risky behav*[tiab] OR irritability[tiab] OR irritable mood*[tiab] OR ((defiant[tiab] OR hostile[tiab]) AND behav*[tiab]) OR aggress*[tiab] OR counteraggress*[tiab] OR anger[tiab] OR "angry outbursts"[tiab] OR provocation*[tiab] OR deceitful*[tiab] OR theft*[tiab] OR stealing*[tiab] OR ((antisocial[tiab] OR anti-social[tiab] OR asocial*[tiab] OR deviant[tiab]) AND (behav*[tiab] OR conduct*[tiab] OR personalit*[tiab] OR reaction*[tiab])) OR social behavior disorder*[tiab] OR social behaviour disorder*[tiab] OR social behavioral disorder*[tiab] OR social behavioural disorder*[tiab] OR misbehav*[tiab] OR misconduct*[tiab] OR delinquency[tiab] OR delinquent*[tiab] OR offender*[tiab] OR callous-unemotional[tiab]) AND (self-control[tiab] OR self manag*[tiab] OR executive function*[tiab] OR "cognitive control"[tiab] OR ((executive[tiab] OR proactive[tiab] OR reactive[tiab]) AND control[tiab]) OR "effortful control"[tiab] OR metacognition[tiab] OR metacognitive[tiab] OR meta-cognit*[tiab] OR ((emotion*[tiab] OR affect[tiab]) AND regulation[tiab]) OR "emotional

control"[tiab] OR (control[tiab] AND emotion*[tiab]) OR "ego control"[tiab] OR "ego undercontrol"[tiab] OR "ego resiliency"[tiab] OR "ego depletion"[tiab] OR autoregulation[tiab] OR self-regulat*[tiab] OR homeostasis[tiab] OR ((cardiac[tiab] OR heart[tiab]) AND (frequenc*[tiab] OR rate*[tiab])) OR "respiratory sinus arrhythmia"[tiab] OR "vagus tone"[tiab] OR "vagal tone"[tiab] OR vagotonus[tiab] OR baroreflex*[tiab] OR "pressoreceptor reflex"[tiab] OR "baroceptor reflex"[tiab] OR "baroreceptor reflex"[tiab] OR "pressor reflex"[tiab] OR "pressure reflex"[tiab] OR "baroreceptor reflex"[tiab] OR ((autonom*[tiab] OR vegetative[tiab]) AND system[tiab]) OR ((adrenergic[tiab] OR sympath*[tiab] OR orthosympath*[tiab]) AND (mechanism[tiab] OR system[tiab]))) AND (adolescent*[tiab] OR adolescence[tiab] OR preadolescen*[tiab] OR pre-adolescen*[tiab] OR teen*[tiab] OR juvenile[tiab] OR youth*[tiab] OR young adult*[tiab]) AND (monitor*[tiab] OR manag*[tiab] OR psychotherap*[tiab] OR therap*[tiab] OR intervention*[tiab] OR treatment*[tiab] OR training*[tiab] OR exercise*[tiab] OR technique*[tiab] OR respiration*[tiab] OR breathing[tiab] OR rehabilitation*[tiab] OR approach*[tiab] OR tool*[tiab] OR remediation*[tiab] OR healing[tiab] OR biofeedback[tiab] OR "bio feed back"[tiab] OR bio-feedback[tiab] OR biofeed-back[tiab]) NOT medline[sb]

APA PsycInfo Ovid SP

APA PsycInfo 1806 to September Week 4 2020
2497 references found

(oppositional defiant disorder/ OR exp impulse control disorders/ OR conduct disorder/ OR antisocial personality disorder/ OR exp behavior problems/ OR behavior disorders/ OR behavioral disinhibition/ OR "inhibition (personality)"/ OR juvenile delinquency/ OR impulsiveness/ OR irritability/ OR aggressive behavior/ OR aggressiveness/ OR theft/ OR antisocial behavior/ OR risk taking/ OR sensation seeking/ OR (externalizing OR externalising OR ODD OR ((oppositional OR defiant) ADJ1 disorder*) OR "intermittent explosive disorder" OR "conduct disorder*" OR ((sociopath* OR psychopath* OR antisocial OR dyssocial) ADJ1 (behav* OR personalit*)) OR sociopathy OR ((aberrant OR deviant OR disturb*) ADJ1 behav*) OR (behav* ADJ1 (aberration OR disorder* OR disturbance OR crisis)) OR runaway OR elopement OR "impulse control disorder*" OR "conduct disorder*" OR ((disruptive OR problem* OR dysfunctional) ADJ3 behav*) OR problembehav* OR impulsiv* OR impulsigenic OR disinhibition OR inhibition OR "sensation seek*" OR risk-taking OR "risk behav*" OR "risky behav*" OR irritability OR "irritable mood*" OR ((defiant OR hostile) ADJ1 behav*) OR aggress* OR counteraggress* OR anger OR "angry outbursts" OR provocation* OR deceitful* OR theft* OR stealing* OR ((antisocial OR anti-social OR asocial* OR deviant) ADJ3 (behav* OR conduct* OR personalit* OR reaction*)) OR "social behav* disorder*" OR misbehav* OR misconduct* OR delinquency OR delinquent* OR offender* OR callous-unemotional OR "callous and unemotional").ab,ti.) AND (self-control/ OR self-regulation/ OR emotional regulation/ OR exp emotional control/ OR executive function/ OR metacognition/ OR ego/ OR homeostasis/ OR cardiovascular reactivity/ OR heart

rate/ OR baroreceptors/ OR autonomic nervous system/ OR (self-control OR "self manag*" OR "executive function*" OR "cognitive control" OR ((executive OR proactive OR reactive) ADJ1 control) OR "effortful control" OR metacognition OR metacognitive OR meta-cognit* OR ((emotion* OR affect) ADJ1 regulation) OR "emotional control" OR (control ADJ3 emotion*) OR "ego control" OR "ego undercontrol" OR "ego resiliency" OR "ego depletion" OR autoregulation OR self-regulat* OR homeostasis OR ((cardiac OR heart) ADJ1 (frequenc* OR rate*)) OR "respiratory sinus arrhythmia" OR "vagus tone" OR "vagal tone" OR vagotonus OR "vagus nerve tone" OR baroreflex* OR "pressoreceptor reflex" OR "baroceptor reflex" OR "baroreceptor reflex" OR "pressor reflex" OR "pressure reflex" OR "baroreceptor reflex" OR ((autonom* OR vegetative) ADJ3 system) OR ((adrenergic OR sympath* OR orthosympath*) ADJ3 (mechanism OR system))).ab,ti.) AND (adolescent attitudes/ OR adolescent psychiatry/ OR adolescent psychology/ OR adolescent psychopathology/ OR adolescent psychotherapy/ OR juvenile delinquency/ OR (adolescent* OR adolescence OR preadolescen* OR pre-adolescen* OR teen* OR juvenile OR youth* OR "young adult*").ab,ti.) AND (exp psychotherapy/ OR exp rehabilitation/ OR exp alternative medicine/ OR biofeedback training/ OR respiration/ OR (monitor* OR manag* OR psychotherap* OR therap* OR intervention* OR treatment* OR training* OR exercise* OR technique* OR respiration* OR breathing OR rehabilitation* OR approach* OR tool* OR remediation* OR healing OR biofeedback OR "bio feed back" OR bio-feedback OR biofeed-back).ab,ti.)

Supplementary file 1: Non-pharmaceutical interventions for self-regulatory failures in adolescents suffering from externalizing symptoms: A scoping review

Cochrane Library Wiley

Cochrane Database of Systematic Reviews, Issue 10 of 12, October 2020, and, Cochrane Central Register of Controlled Trials, Issue 10 of 12, October 2020
986 references found

(externalizing OR externalising OR ODD OR ((oppositional OR defiant) NEXT/1 disorder*) OR "intermittent explosive disorder" OR (conduct NEXT/1disorder*) OR ((sociopath* OR psychopath* OR antisocial OR dyssocial) NEXT/1 (behav* OR personalit*)) OR sociopathy OR ((aberrant OR deviant OR disturb*) NEXT/1 behav*) OR (behav* NEXT/1 (aberration OR disorder* OR disturbance OR crisis)) OR runaway OR elopement OR ("impulse control" NEXT/1 disorder*) OR (conduct NEXT/1 disorder*) OR ((disruptive OR problem* OR dysfunctional) NEAR/3 behav*) OR problembehav* OR impulsiv* OR impulsigenic OR disinhibition OR inhibition OR (sensation NEXT/1 seek*) OR risk-taking OR (risk NEXT/1 behav*) OR (risky NEXT/1 behav*) OR irritability OR (irritable NEXT/1 mood*) OR ((defiant OR hostile) NEXT/1 behav*) OR aggress* OR counteraggress* OR anger OR "angry outbursts" OR provocation* OR deceitful* OR theft* OR stealing* OR ((antisocial OR anti-social OR asocial* OR deviant) NEAR/3 (behav* OR conduct* OR personalit* OR reaction*)) OR (social NEXT/1 behav* NEXT/1 disorder*) OR misbehav* OR misconduct* OR delinquency OR delinquent* OR offender* OR callous-unemotional OR "callous and unemotional"):ab,ti,kw AND (self-control OR (self NEXT/1 manag*) OR (executive NEXT/1 function*) OR "cognitive control" OR ((executive OR proactive OR reactive) NEXT/1 control) OR "effortful control" OR metacognition OR metacognitive OR (meta NEXT/1 cognit*) OR ((emotion* OR affect) NEXT/1 regulation) OR "emotional control" OR (control NEXT/3 emotion*) OR "ego control" OR "ego undercontrol" OR "ego resiliency" OR "ego depletion" OR autoregulation OR (self NEXT/1 regulat*) OR homeostasis OR ((cardiac OR heart) NEXT/1 (frequenc* OR rate*)) OR "respiratory sinus arrhythmia" OR "vagus tone" OR "vagal tone" OR vagotonus OR "vagus nerve tone" OR baroreflex* OR "pressoreceptor reflex" OR "baroceptor reflex" OR "baroreceptor reflex" OR "pressor reflex" OR "pressure reflex" OR "baroreceptor reflex" OR ((autonom* OR vegetative) NEXT/3 system) OR ((adrenergic OR sympath* OR orthosympath*) NEXT/3 (mechanism OR system))):ab,ti,kw AND (adolescent* OR adolescence OR preadolescen* OR (pre NEXT/1 adolescen*) OR teen* OR juvenile OR youth* OR "young adult*"):ab,ti,kw AND (monitor* OR manag* OR psychotherap* OR therap* OR intervention* OR treatment* OR training* OR exercise* OR technique* OR respiration* OR breathing OR rehabilitation* OR approach* OR tool* OR remediation* OR healing OR biofeedback OR "bio feed back" OR (bio NEXT/1 feedback) OR (biofeed NEXT/1 back)):ab,ti,kw

Supplementary file 1: Non-pharmaceutical interventions for self-regulatory failures in adolescents suffering from externalizing symptoms: A scoping review

Web of Science - Core collection

(Science Citation Index Expanded (SCI-EXPANDED) --1900-present ; Social Sciences Citation Index (SSCI) --1900-present ; Arts & Humanities Citation Index (A&HCI) --1975-present ; Conference Proceedings Citation Index- Science (CPCI-S) --1990-present ; Conference Proceedings Citation Index- Social Science & Humanities (CPCI-SSH) --1990-present ; Book Citation Index– Science (BKCI-S) --2005-present ; Book Citation Index– Social Sciences & Humanities (BKCI-SSH) --2005-present ; Emerging Sources Citation Index (ESCI) --2015-present ; Web of Science Core Collection: Chemical Indexes ; Current Chemical Reactions (CCR-EXPANDED) --1985-present ; (Includes Institut National de la Propriete Industrielle structure data back to 1840) ; Index Chemicus (IC) --1993-present)

October 5, 2020
3682 references found

TS=(("externalizing" OR "externalising" OR "ODD" OR (("oppositional" OR "defiant") NEAR/1 disorder*) OR "intermittent explosive disorder" OR "conduct disorder*" OR ((sociopath* OR psychopath* OR "antisocial" OR "dyssocial") NEAR/1 (behav* OR personalit*)) OR "sociopathy" OR (("aberrant" OR "deviant" OR disturb*) NEAR/1 behav*) OR (behav* NEAR/1 ("aberration" OR disorder* OR "disturbance" OR "crisis")) OR "runaway" OR "elopement" OR "impulse control disorder*" OR "conduct disorder*" OR (("disruptive" OR problem* OR "dysfunctional") NEAR/4 behav*) OR problembehav* OR impulsiv* OR "impulsigenic" OR "disinhibition" OR "inhibition" OR "sensation seek*" OR "risk-taking" OR "risk behav*" OR "risky behav*" OR "irritability" OR "irritable mood*" OR (("defiant" OR "hostile") NEAR/1 behav*) OR aggress* OR counteraggress* OR "anger" OR "angry outbursts" OR provocation* OR deceitful* OR theft* OR stealing* OR (("antisocial" OR "anti-social" OR asocial* OR deviant) NEAR/4 (behav* OR conduct* OR personalit* OR reaction*)) OR "social behav* disorder*" OR misbehav* OR misconduct* OR "delinquency" OR delinquent* OR offender* OR "callous-unemotional" OR "callous and unemotional") AND ("self-control" OR "self manag*" OR "executive function*" OR "cognitive control" OR (("executive" OR "proactive" OR "reactive") NEAR/1 "control") OR "effortful control" OR "metacognition" OR "metacognitive" OR meta-cognit* OR ((emotion* OR "affect") NEAR/1 "regulation") OR "emotional control" OR ("control" NEAR/4 emotion*) OR "ego control" OR "ego undercontrol" OR "ego resiliency" OR "ego depletion" OR "autoregulation" OR self-regulat* OR "homeostasis" OR (("cardiac" OR "heart") NEAR/1 (frequenc* OR rate*)) OR "respiratory sinus arrhythmia" OR "vagus tone" OR "vagal tone" OR "vagotonus" OR "vagus nerve tone" OR baroreflex* OR "pressoreceptor reflex" OR "baroceptor reflex" OR "baroreceptor reflex" OR "pressor reflex" OR "pressure reflex" OR "baroreceptor reflex" OR ((autonom* OR "vegetative") NEAR/3 "system") OR (("adrenergic" OR sympath* OR orthosympath*) NEAR/4 ("mechanism" OR "system"))) AND (adolescent* OR "adolescence" OR preadolescen* OR pre-adolescen* OR teen* OR "juvenile" OR youth* OR "young adult*") AND (monitor* OR manag* OR psychotherap* OR therap* OR intervention* OR treatment* OR training* OR exercise* OR technique* OR respiration* OR "breathing" OR rehabilitation* OR approach* OR tool* OR remediation* OR "healing" OR "biofeedback" OR "bio feed back"

OR "bio-feedback" OR "biofeed-back") NOT ((animal* OR plant* OR "rats" OR "mice" OR "pigs") NOT (human* OR patient*)))

ProQuest Dissertations & Theses A&I

August 16, 2019
83 references found
Search options :

- Advanced search
- Search field : anywherer except full text - NOFT
- Doctoral dissertations only

noft((externalizing OR externalising) AND (self-control OR (emotion* AND regulation) OR (emotion* AND control) OR self-regulat*) AND (adolescent* OR adolescence OR preadolescen* OR pre-adolescen* OR teen* OR juvenile OR youth* OR "young adult" OR "young adulthood" OR "young adults") AND (psychotherap* OR therap* OR intervention*))

Dart Europe

August 16, 2019
37 references found

(externalizing OR externalising) AND (adolescent* OR adolescence OR preadolescen* OR pre-adolescen* OR teen* OR juvenile OR youth* OR "young adult" OR "young adulthood" OR "young adults") AND (psychotherap* OR therap* OR intervention*)
