# Supplementary material for: Non-Pharmaceutical Interventions for Self-Regulatory Failures in Adolescents Suffering from Externalizing Symptoms: A Scoping Review
Source: Biomedicines. 2021 Aug 24;9(9):1081. doi: 10.3390/biomedicines9091081 (PMC8466021; doi:10.3390/biomedicines9091081)
Supplement: Supplementary file 1 [file biomedicines-09-01081-s001.zip › Supplementary_file_2.pdf]

Supplementary file 2: Non-pharmaceutical interventions for self-regulatory failures in adolescents suffering from externalizing symptoms: A scoping review

Supplementary Table S1. Included studies (n=239)

**Author, publication date**	**Location**	**Sample size (N)**	**Intervention received (N)**	**Age range**	**Intervention stages**	**Study design***
Alexander & Parsons (1973)	USA	86	46	13 to 16	Indicated	RCT
Apsche et al. (2005)	USA	60	60	*NA*	Treatment	RCT
Arbuthnot & Gordon (1986)	USA	48	24	13 to 17	Selective	RCT
Aronson (1986)	*NA*	1	1	15	Treatment	Single case
Avci & Kelleci (2016)	Turkey	60	30	14 to 15	Selective	RCT
Bachi et al. (2012)	USA	29	14	14 to 18	Selective	Controlled-Trial
Bank et al. (1991)	USA	55	28	*NA*	Indicated	RCT
Barfield & Hutchinson (1990)	USA	10	10	13 to 18	Treatment	Open label
Barnert et al. (2014)	USA	29	16	14 to 18	Indicated	Controlled-Trial
Belknap et al. (2013)	USA	66	66	*NA*	Universal	Open label
Bell, et al. (1983)	USA	45	24	10 to 16	Treatment	RCT
Bögels et al. (2008)	Netherlands	14	14	11 to 17	Treatment	Open label
Borduin et al. (1995)	USA	176	92	12 to 17	Indicated	RCT
Bosworth & Espelage (1998)	USA	119	119	12 to 13	Universal	Open label
Brank et al. (2008)	USA	314	151	12 to 18	Indicated	RCT
Brigham et al. (1985)	USA	79	79	11 to 14	Selective	Open label
Brown & Greenspan (1984)	USA	32	17	14 to 20	Selective	Controlled-Trial
Burt (2018)	USA	50	18	11 to 14	Selective	RCT
Caldwell & Jo (2012)	USA	3	3	14 to 18	Indicated	ABAB design
Cancio et al. (2004)	USA	6	6	11 to 15	Selective	Open label
Cardwell et al. (2019)	Australia	102	51	10 to 16	Selective	RCT
Carpenter & Sandberg (1985)	USA	17	7	14 to 16	Indicated	Controlled-Trial
Carpenter & Sanderberg (1973)	USA	16	10	15 to 16	Indicated	Other
Carr & Punzo (1993)	USA	3	3	13 to 15	Selective	ABAB design
Carroll et al. (2012)	Australia	24	18	12 to 18	Indicated	RCT
Chao-Fernández (2020)	Spain	6	6	16 to 18	Selective	Open label
Claro, Boulanger & Shaw (2015)	Canada	41	28	12 to 17	Selective	Controlled-Trial
Clement (2011)	USA	1	1	12	Treatment	Single case

Supplementary file 2: Non-pharmaceutical interventions for self-regulatory failures in adolescents suffering from externalizing symptoms: A scoping review

**Author, publication date**	**Location**	**Sample size (N)**	**Intervention received (N)**	**Age range**	**Intervention stages**	**Study design***
Coleman, et al. (1992)	USA	39	24	13 to 18	Treatment	RCT
Connell et al. (2007)	USA	998	500	11 to 17	Universal	RCT
Coonerty-Femiano (2008)	USA	14	14	13 to 14	Selective	RCT
Corder et al. (1986)	USA	13	13	13 to 18	Treatment	Open label
Cuenca-Sanchez et al. (2012)	USA	21	11	12 to 15	Selective	RCT
Currie (2004)	Australia	1	1	13	Selective	Single case
Currie & Startup (2012)	Australia	111	60	12 to 15	Selective	RCT
Dangel, et al. (1989)	USA	18	*NA*	10 to 17	Indicated	ABAB design
Davidson et al. (1987)	USA	213	124	*NA*	Indicated	RCT
Davis & Hajicek (1985)	USA	7	7	9 to 15	Treatment	Other
De Fuccio et al. (2009)	USA	52	28	12 to 22	Indicated	Controlled-Trial
Deffenbacher et al. (1996)	USA	120	79	11 to 14	Selective	RCT
Derbyshire et al. (2019)	UK	21	21	16 to 17	Indicated	Open label
Dishion & Andrews (1995)	USA	158	119	10 to 14	Selective	RCT
Dos Santos (2018)	South Africa	6	6	15 to 18	Selective	Qualitative
Down et al. (2011)	UK	25	18	*NA*	Treatment	RCT
Ducharme et al. (2012)	USA	37	18	9 to 17	Treatment	Controlled-Trial
Dykeman (1995)	USA	4	4	13 to 14	Treatment	Other
Elder & Edelstein (1979)	USA	4	4	*NA*	Treatment	ABAB design
Ennis et al. (2015)	USA	44	44	12 to 18	Selective	Open label
Etscheidt (1991)	USA	30	*NA*	12 to 18	Selective	RCT
Feindler (1995)	USA	1	1	13	Selective	Single case
Feindler et al. (1986)	USA	29	21	13 to 18	Treatment	Controlled-Trial
Feindler et al. (1984)	USA	36	18	12 to 15	Selective	RCT
Felver et al. (2020)	USA	23	9	*NA*	Universal	Controlled-Trial
Fishbein et al. (2006)	USA	120	56	*NA*	Selective	RCT
Fosco et al. (2013)	USA	593	386	11 to 14	Universal	RCT
Franco et al. (2016)	Spain	27	13	12 to 19	Universal	RCT

Supplementary file 2: Non-pharmaceutical interventions for self-regulatory failures in adolescents suffering from externalizing symptoms: A scoping review

**Author, publication date**	**Location**	**Sample size (N)**	**Intervention received (N)**	**Age range**	**Intervention stages**	**Study design***
Fung et al. (2016)	USA	19	9	12 to 14	Selective	RCT
Fung et al. (2019)	USA	145	79	13 to 15	Selective	RCT
Gaines & Barry (2008)	USA	6	6	15 to 18	Indicated	ABAB design
Garaigordobil & Peña-Sarrionandia (2015)	Spain	148	83	13 to 16	Universal	RCT
Garrett & Giddings (2014)	USA	5	5	*NA*	Selective	Multiple cases
Garwood et al. (2019)	USA	11	11	13 to 16	Treatment	ABAB design
Gentry & Ostapiuk (1988)	UK	1	1	17	Indicated	Single case
Glick & Goldstein (1987)	USA	60	48	14 to 17	Indicated	RCT
Glomb & West (1990)	USA	2	2	*NA*	Selective	Multiple cases
Goldstein et al. (2018)	USA	57	29	14 to 20	Indicated	RCT
Gómez et al. (2014)	Spain	5	5	15 to 17	Treatment	Other
Gonzales et al. (2012)	USA	516	338	*NA*	Universal	RCT
Gottfredson et al. (2002)	USA	188	90	12 to 14	Universal	RCT
Gregory et al. (1997)	*NA*	3	3	13 to 14	Selective	ABAB design
Gross et al. (1980)	USA	10	10	12 to 16	Indicated	Open label
Guerra (1990)	USA	120	80	15 to 18	Indicated	RCT
Hains (1984)	USA	4	4	14 to 17	Indicated	ABAB design
Hains (1989)	USA	4	4	15 to 17	Indicated	Open label
Hains & Hains (1988)	USA	5	5	15 to 17	Indicated	ABBA crossover
Hanselman (2001)	USA	7	7	*NA*	Indicated	ABAB design
Hansen et al. (2013)	USA	3	3	7 to 12	Selective	Multiple cases
Harvey, et al (2020)	USA	19	19	10 to 17	Selective	Open label
Havighurst et al. (2015)	Australia	212	113	10 to 13	Universal	RCT
Hawkins et al. (1991)	USA	141	69	11 to 18	Indicated	RCT
Hay et al. (2010)	USA	671	338	12 to 15	Selective	RCT
Heaton et al. (1976)	USA	46	32	13 to 14	Selective	Controlled-Trial
Hein et al. (2020) – Study 1	Germany	476	305	16.03	Indicated	RCT
Hein et al. (2020) – Study 2	Germany	476	305	16.03	Indicated	Open label
Henggeler et al. (1992)	USA	56	33	*NA*	Indicated	RCT

**Author, publication date**	**Location**	**Sample size (N)**	**Intervention received (N)**	**Age range**	**Intervention stages**	**Study design***
Hentges et al. (2020)	USA	731	367	*NA*	Universal	RCT
Hilyer et al. (1982)	USA	43	23	15 to 18	Indicated	RCT
Himelstein et al. (2011)	USA	32	32	14 to 18	Indicated	Open label
Hobbs & Holt (1976)	USA	125	*NA*	12 to 15	Indicated	Controlled-Trial
Hogue et al. (2017)	USA	205	104	12 to 18	Treatment	RCT
Holmqvist et al. (2009)	Sweden	57	26	16 to 19	Indicated	Controlled-Trial
Hoogsteder et al. (2018)	Netherlands	91	63	16 to 21	Indicated	Controlled-Trial
Hornsveld et al. (2008)	Netherlands	142	*NA*	13 to 20	Treatment	Open label
Hornsveld et al. (2015)	Netherlands	62	*NA*	15 to 21	Indicated	Controlled-Trial
Houck et al. (2016)	USA	420	222	12 to 14	Selective	ABBA crossover
Hovell et al. (2001)	USA	29	29	13 to 18	Indicated	Controlled-Trial
Huey & Rank (1984)	USA	48	24	13 to 15	Selective	RCT
Huey et al. (2000)	USA	114	54	*NA*	Indicated	RCT
Jamali et al. (2016)	Iran	100	50	*NA*	Universal	RCT
Jones et al. (2004)	USA	35	24	11 to 16	Indicated	Controlled-Trial
Kaffman (1968)	Israel	32	32	8 to 15	Treatment	Multiple cases
Kahn & McFarland (1973)	USA	47	47	*NA*	Indicated	Open label
Kanchibhotla et al. (2021)	India	454	237	13 to 17	Universal	Other
Kappes & Thompson (1985)	USA	26	12	15 to 18	Indicated	RCT
Kastner (1998)	USA	11	11	*NA*	Treatment	Open label
Kauser & Pinquart (2019)	Pakistan	110	50	*NA*	Selective	RCT
Kaya & Buzlu (2016)	Turkey	65	32	14 to 17	Indicated	Controlled-Trial
Keiley (2007)	USA	73	73	13 to 18	Indicated	Open label
Kellner &. Bry (1999)	USA	7	7	14 to 18	Selective	Open label
Kellner et al. (2002)	USA	46	27	12 to 16	Selective	Controlled-Trial
Kellner et al. (2003)	USA	46	27	12 to 16	Selective	Controlled-Trial
Kethineni & Braithwaite (2010)	USA	172	86	11 to 18	Indicated	Controlled-Trial
Kifer et al. (1974)	USA	3	3	13 to 17	Selective	ABAB design
Kimber et al. (2008)	Sweden	1417	1028	7 to 16	Universal	Controlled-Trial

**Author, publication date**	**Location**	**Sample size (N)**	**Intervention received (N)**	**Age range**	**Intervention stages**	**Study design***
Langeveld et al. (2012)	Norway	112	112	*NA*	Universal	RCT
Larson (1992)	USA	37	22	*NA*	Selective	RCT
Larson et al. (1998)	USA	*NA*	*NA*	*NA*	Treatment	Open label
Lazarus (1993)	USA	18	*NA*	11 to 13	Selective	Open label
Le & Proulx (2015)	Hawaii	36	36	14 to 18	Indicated	Open label
Lee et al. (1979)	Canada	26	7	14 to 15	Selective	RCT
Leonard et al. (2013)	USA	264	147	16 to 18	Indicated	RCT
Leve et al. (2005)	USA	81	37	13 to 17	Indicated	RCT
Lök et al. (2018)	Turkey	60	30	*NA*	Selective	RCT
Lombas et al. (2019)	Spain	524	156	12 to 17	Universal	Controlled-Trial
Long & Sherer (1984)	USA	27	9	12 to 17	Indicated	Controlled-Trial
Lutz (2014)	USA	*NA*	*NA*	*NA*	Treatment	Single case
MacMahon & Gross (1988)	USA	69	32	14 to 18	Indicated	RCT
Marco et al. (2013)	*NA*	2	2	12 to 18	Treatment	Multiple cases
Marshall & Heward (1979)	*NA*	8	8	16 to 17	Indicated	Open label
Martin & Johnson (2005)	USA	7	7	14 to 17	Indicated	Open label
Martsch (2005)	USA	65	34	13 to 18	Indicated	RCT
Marvit et al. (1974)	Hawaii	44	23	*NA*	Indicated	RCT
Mason et al. (2010)	USA	5	5	12 to 14	Selective	Open label
Mason et al. (2016)	USA	321	118	*NA*	Universal	Other
Mathur & Rutherford (1994)	USA	9	9	13 to 17	Indicated	ABAB design
Maya et al. (2019)	Spain	216	109	11 to 17	Selective	Controlled-Trial
Mazerolle et al. (2017)	Australia	102	51	10 to 16	Selective	RCT
Mazerolle et al. (2017)	Australia	102	51	10 to 16	Selective	RCT
Mazerolle et al. (2019)	Australia	102	51	10 to 16	Indicated	RCT
Mazerolle et al. (2020)	Australia	102	51	*NA*	Universal	RCT
McAllister et al. (1969)	USA	51	25	16 to 19	Universal	Controlled-Trial
McCarthy-Tucker et al. (1999)	USA	8	8	*NA*	Indicated	Open label
McCraty et al. (1999)	USA	92	62	12 to 14	Selective	RCT

**Author, publication date**	**Location**	**Sample size (N)**	**Intervention received (N)**	**Age range**	**Intervention stages**	**Study design***
McCullough et al. (1977)	USA	1	1	16	Treatment	Single case
McMahon & Washbur (2003)	USA	149	149	11 to 14	Selective	Open label
McNamara & Heard (1976)	UK	20	20	13 to 14	Universal	RCT
McNamara & Jolly (1990)	UK	15	*NA*	12 to 13	Universal	Open label
McWirther & Page (1999)	USA	57	19	16 to 19	Selective	Controlled-Trial
Minkos et al. (2018)	USA	2	2	15 to 16	Selective	Open label
Minor & Elrod (1994)	USA	45	22	12 to 17	Indicated	RCT
Mohammadi et al. (2017)	Iran	8	8	14 to 18	Indicated	Open label
Moneta & Rousseau (2008)	Canada	20	20	13 to 16	Selective	Qualitative
Moretti & Obsuth (2009)	Canada	329	*NA*	*NA*	Treatment	Controlled-Trial
Mutiso et al. (2018)	Kenya	630	171	11 to 18	Selective	RCT
Neel & De Bruler (1979)	USA	73	55	12 to 15	Selective	Open label
Nelson Gray et al. (2006)	USA	32	32	10 to 15	Treatment	Open label
Nickel et al. (2005)	*NA*	87	44	16 to 18	Selective	RCT
Niles (1986)	USA	59	19	13 to 15	Indicated	RCT
Ninness et al. (1995)	USA	4	4	14 to 15	Selective	ABAB design
Nugent et al. (1999)	USA	522	*NA*	11 to 17	Indicated	Open label
Nugent et al. (1997)	USA	102	9	12 to 18	Indicated	Controlled-Trial
Ollendick & Hersen (1979)	USA	27	*NA*	13 to 16	Indicated	RCT
Oruche et al. (2018)	USA	20	11	12 to 18	Treatment	RCT
Panosky & Shelton (2015)	USA	6	6	16 to 17	Indicated	ABAB design
Pardo et al. (2020)	Spain	20	*NA*	14 to 17	Treatment	Qualitative
Patrick & Rich (2004)	*NA*	8	8	*NA*	Indicated	Single case
Patterson (1974)	USA	41	41	*NA*	Selective	Open label
Pazaratz (1998)	Canada	1	1	15	Treatment	Single case
Peterson et al. (1999)	USA	29	29	12 to 14	Selective	Open label
Phillips et al. (1971)	USA	6	6	12 to 15	Indicated	ABAB design
Pop-Jordanova (2009)	Macedonia	74	59	*NA*	Treatment	Controlled-Trial
Presley & Hughes (2000)	USA	4	4	14 to 17	Selective	Open label

**Author, publication date**	**Location**	**Sample size (N)**	**Intervention received (N)**	**Age range**	**Intervention stages**	**Study design***
Puskar et al. (2015)	USA	179	93	14 to 18	Universal	RCT
Quinn & Van Dyke (2004)	USA	362	267	*NA*	Indicated	Controlled-Trial
Ramadoss & Bose (2010)	USA	70	70	16 to 17	Indicated	Open label
Rapp-Paglicci et al. (2011)	USA	108	108	15 to 18	Indicated	Open label
Redondo et al. (2012)	Spain	28	17	15 to 20	Indicated	Controlled-Trial
Rowlands et al. (2020)	USA	14	14	13 to 17	Indicated	Open label
Richman & Harper (1979)	USA	54	54	*NA*	Indicated	Open label
Rickson & Watkins (2003)	New Zealand	15	11	11 to 15	Treatment	RCT
Rizzo et al. (2020)	USA	119	59	*NA*	Universal	RCT
Robinson, et al. (2002)	USA	41	22	11 to 15	Selective	RCT
Rodríguez et al. (2016)	Costa Rica	28	28	*NA*	Selective	Qualitative
Rogevich & Perin (2008)	USA	63	30	13 to 16	Treatment	Controlled-Trial
Rohde et al. (2004)	USA	138	46	12 to 25	Indicated	RCT
Ronen (2004)	Israel	1	1	12	Treatment	Single case
Ronen & Rosenbaum (2009)	Israel	447	167	14 to 15	Selective	Controlled-Trial
Rosenberg (2004)	USA	1347	1084	13 to 14	Universal	Controlled-Trial
Ruttledge & Petrides (2011)	Ireland	22	22	13 to 14	Selective	Open label
Santogrossi et al. (1973)	USA	9	9	12 to 15	Treatment	ABAB design
Sanz de Acedo & Iriarte (2001)	Spain	109	50	14 to 16	Selective	RCT
Scherer et al. (1994)	USA	55	23	11 to 17	Indicated	RCT
Schippers et al. (2020)	Netherlands	25	11	13 to 23	Treatment	RCT
Schlichter & Horan (1981)	USA	38	10	13 to 18	Indicated	RCT
Schloss et al. (1983)	USA	1	1	18	Treatment	Single case
Schuurmans et al. (2018)	Netherlands	37	18	*NA*	Treatment	RCT
Seckman et al. (2016)	USA	*NA*	*NA*	12 to 17	Treatment	Open label
Seivert et al. (2018)	USA	138	83	13 to 18	Indicated	RCT
Shahbazi et al. (2017)	IRAN	30	15	15 to 18	Selective	RCT
Sharry & Owens (2000)	UK	6	6	13 to 16	Selective	Multiple cases
Shivrattan (1988)	Canada	43	14	15 to 17	Indicated	RCT

**Author, publication date**	**Location**	**Sample size (N)**	**Intervention received (N)**	**Age range**	**Intervention stages**	**Study design***
Singh et al. (2007)	USA	3	3	13 to 14	Treatment	Open label
Smaller (2012)	USA	15	2	*NA*	Selective	Multiple cases
Smith et al. (2017)	USA	152	83	11 to 15	Selective	RCT
Smith et al. (1998)	USA	4	4	13 to 15	Selective	ABAB design
Synder & Shapiro (1997)	USA	3	3	14 to 15	Selective	Open label
Snyder & White (1979)	USA	15	5	14 to 17	Indicated	RCT
Snyder et al. (1999)	USA	50	25	*NA*	Treatment	RCT
Sosa-Rubi et al. (2017)	Mexico	885	381	*NA*	Universal	Controlled-Trial
Spence & Marzillier (1981)	UK	76	32	10 to 16	Indicated	RCT
Stormshak et al. (2005)	USA	584	584	*NA*	Universal	Open label
Sutton et al. (2013)	New Zealand	20	20	*NA*	Treatment	Qualitative
Syzmanski et al. (2018)	USA	73	43	*NA*	Indicated	RCT
Teeter et al. (2000)	USA	17	8	*NA*	Selective	RCT
Thoder & Cautilli (2011)	USA	39	39	14 to 17	Indicated	Open label
Titilayo & Aderanti (2012)	Nigeria	72	36	9 to 16	Indicated	Controlled-Trial
Trajkovic et al. (2020)	Italy	107	56	*NA*	Universal	RCT
Trulson (1986)	USA	34	15	13 to 17	Indicated	Controlled-Trial
Trupin et al. (2011)	USA	274	105	12 to 19	Indicated	Controlled-Trial
Twemlow & Sacco (1998)	USA	3	3	10 to 16	Indicated	Multiple cases
Tyson (2002)	USA	11	5	*NA*	Indicated	RCT
Umbach et al. (2018)	USA	197	109	16 to 18	Indicated	RCT
Uzunoglu & Baysan-Arabaci (2017)	Turkey	16	8	12 to 18	Treatment	Controlled-Trial
Valliant et al. (1995)	Canada	39	29	16 to 18	Indicated	Controlled-Trial
Van Bockstaele et al. (2020)	Netherlands	39	19	12 to 16	Selective	RCT
Van Manen et al. (2004)	Netherlands	97	42	9 to 13	Treatment	RCT
Van Vliet et al. (2017)	Canada	28	28	12 to 17	Treatment	RCT
Van Wijk-Herbrink et al. (2017)	Netherlands	4	4	15 to 17	Treatment	Multiple cases
Wainryb et al. (2018)	USA	241	114	8 to 17	Universal	RCT
West et al. (2017)	Australia	112	56	12 to 18	Treatment	Open label

Supplementary file 2: Non-pharmaceutical interventions for self-regulatory failures in adolescents suffering from externalizing symptoms: A scoping review

**Author, publication date**	**Location**	**Sample size (N)**	**Intervention received (N)**	**Age range**	**Intervention stages**	**Study design***
Whitfield (1999)	USA	28	8	*NA*	Selective	Controlled-Trial
Williams & Akamatsu (1978)	USA	30	*NA*	*NA*	Indicated	RCT
Wodarski et al. (1979)	USA	40	21	*NA*	Indicated	RCT
Wolff & Ollendick (2012)	USA	5	5	11 to 14	Treatment	ABAB design
Wongtongkam et al. (2014)	Thailand	96	40	*NA*	Universal	Controlled-Trial
Yektatalab et al. (2017)	Iran	140	70	13 to 15	Universal	RCT
Zapolski & Smith (2017)	USA	53	53	12 to 14	Selective	Open label
Zhou et al. (2018)	Singapore	156	*NA*	*NA*	Indicated	Open label
Zivin et al. (2001)	USA	60	32	11 to 14	Selective	ABBA crossover

*Study design: RCT = Randomized controlled trial

References

1. Alexander, J.F.; Parsons, B.V. Short-term behavioral intervention with delinquent families: impact on family process and recidivism. *Journal of Abnormal Psychology* **1973**, *81*, 219-225, doi:10.1037/h0034537.
2. Apsche, J.A.; Bass, C.K.; Siv, A.M.; Matteson, S.C. An Empirical "Real World" Comparison of Two Treatments wit Aggressive Adolescent Males. *International Journal of Behavioral and Consultation Therapy* **2005**, *1*, 239-251, doi:10.1037/h0100748.
3. Arbuthnot, J.; Gordon, D.A. Behavioral and cognitive effects of a moral reasoning development intervention for high-risk behavior-disordered adolescents. *Journal of Consult Clinical Psychology* **1986**, *54*, 208-216, doi:10.1037//0022-006x.54.2.208.
4. Aronson, D.M. The adolescent as hypnotist: Hypnosis and self-hypnosis with adolescent psychiatric inpatients. *American Journal of Clinical Hypnosis* **1986**, *28*, 163-169.
5. Avci, D.; Kelleci, M. Effects of the Anger Coping Programme based on cognitive behavioural techniques on adolescents' anger, aggression and psychological symptoms. *International Journal of Nursing Practice* **2016**, *22*, 189-196.
6. Bachi, K.; Terkel, J.; Teichman, M. Equine-facilitated psychotherapy for at-risk adolescents: the influence on self-image, self-control and trust. *Clinical Child Psychology and Psychiatry* **2012**, *17*, 298-312, doi:10.1177/1359104511404177.
7. Bank, L.; Marlowe, J.H.; Reid, J.B.; Patterson, G.R.; Weinrott, M.R. A comparative evaluation of parent-training interventions for families of chronic delinquents. *Journal of Abnormal Child Psychology* **1991**, *19*, 15-33, doi:10.1007/BF00910562.
8. Barfield, C.K.; Hutchinson, M.A. Observations on Adolescent Anger and an Anger Control Group in Residential and Day Treatment. *Residential Treatment for Children & Youth* **1990**, *7*, 45-58.
9. Barnert, E.S.; Himelstein, S.; Herbert, S.; Garcia-Romeu, A.; Chamberlain, L.J. Innovations in Practice: Exploring an intensive meditation intervention for incarcerated youth. *Child and Adolescent Mental Health* **2014**, *19*, 69-73.
10. Belknap, R.A.; Haglund, K.; Felzer, H.; Pruszynski, J.; Schneider, J. A Theater Intervention to Prevent Teen Dating Violence for Mexican-American Middle School Students. *Journal of Adolescent Health* **2013**, *53*, 62-67.
11. Bell, C.R.; Mundy, P.; Quay, H.C. Modifying Impulsive Responding in Conduct-Disordered Institutionalized Boys. *Psychological Reports* **1983**, *52*, 307-310.

12. Bögels, S.; Hoogstad, B.; Van Dun, L.; De Schutter, S.; Restifo, K. Mindfulness Training for Adolescents with Externalizing Disorders and their Parents. *Behavioural and Cognitive Psychotherapy* **2008**, *36*, 193-209.
13. Borduin, C.M.; Mann, B.J.; Cone, L.T.; Henggeler, S.W.; Fucci, B.R.; Blaske, D.M.; Williams, R.A. Multisystemic treatment of serious juvenile offenders: long-term prevention of criminality and violence. *Journal of Consulting and Clinical Psychology* **1995**, *63*, 569-578, doi:10.1037//0022-006x.63.4.569.
14. Bosworth, K.; Espelage, D.; DuBay, T. A computer-based violence prevention intervention for young adolescents: Pilot study. *Adolescence* **1998**, *33*, 785-795.
15. Brank, E.M.; Lane, J.; Turner, S.; Fain, T.; Sehgal, A. An Experimental Juvenile Probation Program: Effects on Parent and Peer Relationships. *Crime & Delinquency* **2008**, *54*, 193-224.
16. Brigham, T.A.; Hopper, C.; Hill, B.; De Armas, A.; Newsom, P. A Self-Management Program for Disruptive Adolescents in the School: A Clinical Replication Analysis. *Behavior Therapy* **1985**, *16*, 99-115.
17. Brown, G.M.; Greenspan, S. Effect of Social Foresight Training on the School Adjustment of High-Risk Youth. *Child Study Journal* **1984**, *14*, 61-77.
18. Burt, I. Leadership-Driven Anger Management Groups for Adolescents: Do They Really Work? *The Journal for Specialists in Group Work* **2018**, *43*, 57-80.
19. Caldwell, S.; Joseph, L.M. Helping female juveniles improve their on-task behavior and academic performance using a self-management procedure in a correctional facility. *Contemporary School Psychology* **2012**, *16*, 61-74.
20. Cancio, E.J.; West, R.P.; Young, K.R. Improving Mathematics Homework Completion and Accuracy of Students with EBD Through Self-Management and Parent Participation. *Journal of Emotional and Behavioral Disorders* **2004**, *12*, 9-22.
21. Cardwell, S.M.; Mazerolle, L.; Bennett, S.; Piquero, A.R. Changing the Relationship Between Impulsivity and Antisocial Behavior: The Impact of a School Engagement Program. *Crime & Delinquency* **2019**, *65*, 1076-1101.
22. Carpenter, P.; Sandberg, S. "The things inside:" psychodrama with delinquent adolescents. *Psychotherapy: Theory, Research and Practice* **1973**, *10*, 245-247.
23. Carpenter, P.; Sandberg, S. Further Psychodrama with Delinquent Adolescents. *Adolescence* **1985**, *20*, 599-604.

24. Carr, S.C.; Punzo, R.P. The Effects of Self-Monitoring of Academic Accuracy and Productivity on the Performance of Students with Behavioral Disorders. *Behavioral Disorders* **1993**, *18*, 241-250.

25. Carroll, A.; Ashman, A.; Hemingway, F.; Bower, J.; Houghton, S. A Preliminary Evaluation of Mindfields: A Self-Regulatory Cognitive Behavioural Program for School-Aged Adolescent Offenders. *The Australian Educational and Developmental Psychologist* **2012**, *29*, 81-94.

26. Chao-Fernandez, R.; Gisbert-Caudeli, V.; Vazquez-Sanchez, R. Emotional Training and Modification of Disruptive Behaviors through Computer-Game-Based Music Therapy in Secondary Education. *Appl Sci-Basel* **2020**, *10*, doi:10.3390/app10051796.

27. Claro, A.; Boulanger, M.-M.; Shaw, S.R. Targeting Vulnerabilities to Risky Behavior: an Intervention for Promoting Adaptive Emotion Regulation in Adolescents. *Contemporary School Psychology* **2015**, *19*, 330-339.

28. Clement, P.W. A Strengths-Based, Skill-Building, Integrative Approach to Treating Conduct Problems in a 12-Year-Old Boy: Rafael's Story. *Pragmatic Case Studies in Psychotherapy* **2011**, *7*, 351-398.

29. Coleman, M.; Pfeiffer, S.; Oakland, T. Aggression Replacement Training with Behaviorally Disordered Adolescents. *Behavioral Disorders* **1992**, *18*, 54-66.

30. Connell, A.M.; Dishion, T.J.; Yasui, M.; Kavanagh, K. An Adaptive Approach to Family Intervention: Linking Engagement in Family-Centered Intervention to Reductions in Adolescent Problem Behavior. *Journal of Consulting and Clinical Psychology* **2007**, *75*, 568–579.

31. Coonerty-Femiano, A. Developing and evaluating an anger management intervention for boys: What does gender have to do with it? *Dissertation* **2008**, *69*, 5771.

32. Corder, B.F.; Whiteside, R.; Haizlip, T. Biofeedback, cognitive training and relaxation techniques as multimodal adjunct therapy for hospitalized adolescents: A pilot study. *Adolescence* **1986**, *21*, 339-346.

33. Cuenca-Sanchez, Y.; Mastropieri, M.A.; Scruggs, T.E.; Kidd, J.K. Teaching Students with Emotional and Behavioral Disorders to Self-advocate through Persuasive Writing. *Exceptionality* **2012**, *20*, 71-93.

34. Currie, M. Doing Anger Differently: A Group Percussion Therapy for Angry Adolescent Boys. *International Journal Of Group Psychotherapy* **2004**, *54*, 275-294.

35. Currie, M.; Startup, M. Doing Anger Differently: Two controlled trials of percussion group psychotherapy for adolescent reactive aggression. *Journal of Adolescence* **2012**, *35*, 843-853.
36. Dangel, R.F.; Deschner, J.P.; Rasp, R.R. Anger Control Training for Adolescents in Residential-Treatment. *Behavior Modification* **1989**, *13*, 447-458.
37. Davidson, W.S.; Redner, R.; Blakely, C.H.; Mitchell, C.M.; Emshoff, J.G. Diversion of Juvenile Offenders: An Experimental Comparison. *Journal of Consulting and Clinical Psychology* **1987**, *55*, 68-75.
38. Davis, R.W.; Hajicek, J.O. Effects of Self-Instructional Training and Strategy Training on a Mathematics Task with Severely Behaviorally Disordered Students. *Behavioral Disorders* **1985**, *10*, 211-218.
39. De Fuccio, M.; Kuhn, D.; Udell, W.; Callender, K. Developing Argument Skills in Severely Disadvantaged Adolescent Males in a Residential Correctional Setting. *Applied Developmental Science* **2009**, *13*, 30-41.
40. Deffenbacher, J.L.; Lynch, R.S.; Oetting, E.R.; Kemper, C.C. Anger Reduction in Early Adolescents. *Journal of Counseling Psychology* **1996**, *43*, 149-157.
41. Derbyshire, J.M.; Tarrant, E.; Fitter, R.; Gibson, R.A. Evaluating treatment outcomes for young people participating in a high-intensity therapeutic violence intervention in the English Youth Custody Service. *Legal and Criminological Psychology* **2019**, *24*, 162-178.
42. Dishion, T.J.; Andrews, D.W. Preventing escalation in problem behaviors with high-risk young adolescents: immediate and 1-year outcomes. *Journal of consulting and Clinical Psychology* **1995**, *63*, 538-548, doi:10.1037//0022-006x.63.4.538.
43. Dos Santos, A. Empathy and Aggression in Group Music Therapy with Teenagers: A Descriptive Phenomenological Study. *Music Therapy Perspectives* **2018**, *37*, 14-27.
44. Down, R.; Willner, P.; Watts, L.; Griffiths, J. Anger management groups for adolescents: A mixed-methods study of efficacy and treatment preferences. *Clinical Child Psychology and Psychiatry* **2011**, *16*, 33-52.
45. Ducharme, P.; Wharff, E.; Kahn, J.; Hutchinson, E.; Logan, G.; Waber, D.; Holland, J.; Gosselin, G.; Gonzalez-Heydrich, J. Augmenting Anger Control Therapy with a Videogame Requiring Emotional Control: A Pilot Study on an Inpatient Psychiatric Unit. *Adolescent Psychiatry* **2012**, *2*, 323-332.

46. Dykeman, B.F. The Social Cognitive Treatment of Anger and Aggression in Four Adolescents with Conduct Disorder. *Journal of Instructional Psychology* **1995**, *22*, 194-200.
47. Elder, J.P.; Edelstein, B.A. Adolescent Psychiatric Patients: Modifying Aggressive Behavior with Social Skills Training. *Behavior Modification* **1979**, *3*, 161-178.
48. Ennis, R.P.; Jolivette, K.; Terry, N.P.; Fredrick, L.D.; Alberto, P.A. Classwide Teacher Implementation of Self-Regulated Strategy Development for Writing with Students with E/BD in a Residential Facility. *Journal of Behavioral Education* **2015**, *24*, 88-111.
49. Etscheidt, S. Reducing aggressive behavior and improving self-control: A cognitive-behavioral training program for behaviorally disordered adolescents. *Behavioral Disorders* **1991**, *16*, 107-115.
50. Feindler, E.L. Ideal treatment package for children and adolescents with anger disorders. *Issues in Comprehensive Pediatric Nursing* **1995**, *18*, 233-260.
51. Feindler, E.L.; Ecton, R.B.; Kingsley, D.; Dubey, D.R. Group anger-control training for institutionalized psychiatric male adolescents. *Behavior Therapy* **1986**, *17*, 109-123.
52. Feindler, E.L.; Marriott, S.A.; Iwata, M. Group anger control training for junior high school delinquents. *Cognitive Therapy and Research* **1984**, *8*, 299-311.
53. Felver, J.C.; Razza, R.; Morton, M.L.; Clawson, A.J.; Mannion, R.S. School-based yoga intervention increases adolescent resilience: a pilot trial. *Journal of Child & Adolescent Mental Health* **2020**, *32*, 1-10, doi:10.2989/17280583.2019.1698429.
54. Fishbein, D.H.; Hyde, C.; Eldreth, D.; Paschall, M.J.; Hubal, R.; Das, A.; Tarter, R.; Ialongo, N.; Hubbard, S.; Yung, B. Neurocognitive skills moderate urban male adolescents' responses to preventive intervention materials. *Drug and Alcohol Dependence* **2006**, *82*, 47-60.
55. Fosco, G.M.; Frank, J.L.; Stormshak, E.A.; Dishion, T.J. Opening the "Black Box": family check-up intervention effects on self-regulation that prevents growth in problem behavior and substance use. *Journal of School Psychology* **2013**, *51*, 455-468.
56. Franco, C.; Amutio, A.; Lopez-Gonzalez, L.; Oriol, X.; Martinez-Taboada, C. Effect of a Mindfulness Training Program on the Impulsivity and Aggression Levels of Adolescents with Behavioral Problems in the Classroom. *Frontiers in Psychology* **2016**, *7*, 1385.
57. Fung, J.; Guo, S.S.; Jin, J.; Bear, L.; Lau, A. A Pilot Randomized Trial Evaluating a School-Based Mindfulness Intervention for Ethnic Minority Youth. *Mindfulness* **2016**, *7*, 819-828.

58. Fung, J.; Kim, J.J.; Jin, J.; Chen, G.; Bear, L.; Lau, A.S. A Randomized Trial Evaluating School-Based Mindfulness Intervention for Ethnic Minority Youth: Exploring Mediators and Moderators of Intervention Effects. *Journal of Abnormal Child Psychology* **2019**, *47*, 1-19.

59. Gaines, T.; Barry, L.M. The effect of a self-monitored relaxation breathing exercise on male adolescent aggressive behavior. *Adolescence* **2008**, *43*, 291-302.

60. Garaigordobil, M.; Pena-Sarrionandia, A. Effects of an emotional intelligence program in variables related to the prevention of violence. *Frontiers in Psychology* **2015**, *6*, 743.

61. Garrett, K.J.; Giddings, K. Improving impulse control: using an evidence-based practice approach. *Journal of Evidence-Based Social Work* **2014**, *11*, 73-83.

62. Garwood, J.D.; Werts, M.G.; Mason, L.H.; Harris, B.; Austin, M.B.; Ciullo, S.; Magner, K.; Koppenhaver, D.A.; Shin, M. Improving Persuasive Science Writing for Secondary Students With Emotional and Behavioral Disorders Educated in Residential Treatment Facilities. *Behavioral Disorders* **2019**, *44*, 227-240.

63. Gentry, M.; Ostapiuk, E.B. Management of violence in a youth treatment centre. *Issues in Criminological & Legal Psychology* **1988**, *12*, 58-68.

64. Glick, B.; Goldstein, A.P. Aggression Replacement Training. *Journal of Conseling and Development* **1987**, *65*, 356-362.

65. Glomb, N.; West, R.P. Teaching behaviorally disordered adolescents to use self-management skills for improving the completeness, accuracy, and neatness of creative writing homework assignments. *Behavioural Disorders* **1990**, *15*, 233-242.

66. Goldstein, N.E.S.; Giallella, C.L.; Haney-Caron, E.; Peterson, L.; Serico, J.; Kemp, K.; Romaine, C.R.; Zelechoski, A.D.; Holliday, S.B.; Kalbeitzer, R.; et al. Juvenile Justice Anger Management (JJAM) Treatment for Girls: Results of a randomized controlled trial. *Psychological Services* **2018**, *15*, 386-397.

67. Gomez, M.J.; Luciano, C.; Paez-Blarrina, M.; Ruiz, F.J.; Valdivia-Salas, S.; Gil-Luciano, B. Brief ACT protocol in at-risk adolescents with conduct disorder and impulsivity. *International Journal of Psychology and Psychological Therapy* **2014**, *14*, 307-332.

68. Gonzales, N.A.; Dumka, L.E.; Millsap, R.E.; Gottschall, A.; McClain, D.B.; Wong, J.J.; German, M.; Mauricio, A.M.; Wheeler, L.; Carpentier, F.D.; et al. Randomized trial of a broad preventive intervention for Mexican American adolescents. *Journal of consulting and clinical psychology* **2012**, *80*, 1-16, doi:10.1037/a0026063.

69. Gottfredson, G.D.; Jones, E.M.; Gore, T.W. Implementation and evaluation of a cognitive-behavioral intervention to prevent problem behavior in a disorganized school. *Prevention Science* **2002**, *3*, 43-56, doi:10.1023/a:1014671310038.
70. Gregory, K.M.; Kehle, T.J.; McLoughlin, C.S. Generalization and maintenance of treatment gains using self-management procedures with behaviorally disordered adolescents. *Psychological Reports* **1997**, *80*, 683-690.
71. Gross, A.M.; Brigham, T.A.; Hopper, C.; Bologna, N.C. Self-Management and Social Skills Training - a Study with Pre-Delinquent and Delinquent Youths. *Criminal Justice And Behavior* **1980**, *7*, 161-184.
72. Guerra, N.G.; Slaby, R.G. Cognitive Mediators of Aggression in Adolescent Offenders: 2. Intervention. *Developmental Psychology* **1990**, *26*, 269-277.
73. Hains, A.A. A preliminary attempt to teach the use of social problem-solving skills to delinquents. *Child Study Journal* **1984**, *14*, 271-285.
74. Hains, A.A. An anger-control intervention with aggressive delinquent youths. *Behavioral Residential Treatment* **1989**, *4*, 213-230.
75. Hains, A.A.; Hains, A.H. Cognitive-behavioral training of problem-solving and impulse-control with delinquent adolescents. *Journal of Offender Counseling* **1988**, *12*, 95-113.
76. Hanselman, J.L. Coping skills interventions with adolescents in anger management using animals in therapy. *Journal of Child and Adolescent Group Therapy* **2001**, *11*, 159-195.
77. Hansen, B.D.; Wills, H.P.; Kamps, D.M.; Greenwood, C.R. The Effects of Function-Based Self-Management Interventions on Student Behavior. *Journal of Emotional and Behavioral Disorders* **2013**, *22*, 149-159.
78. Harvey, C.; Jedlicka, H.; Martinez, S. A Program Evaluation: Equine-Assisted Psychotherapy Outcomes for Children and Adolescents. *Child and Adolescent Social Work Journal* **2020**.
79. Havighurst, S.S.; Kehoe, C.E.; Harley, A.E. Tuning in to teens: Improving parental responses to anger and reducing youth externalizing behavior problems. *Journal of Adolescence* **2015**, *42*, 148-158.
80. Hawkins, J.D.; Jenson, J.M.; Catalano, R.F.; Wells, E.A. Effects of a skills training intervention with juvenile delinquents. *Research on Social Work Practice* **1991**, *1*, 107-121.

81. Hay, C.; Meldrum, R.; Forrest, W.; Ciaravolo, E. Stability and change in risk seeking: Investigating the effects of an intervention program. *Youth Violence and Juvenile Justice* **2010**, *8*, 91-106.
82. Heaton, R.C.; Safer, D.J.; Allen, R.P.; Spinnato, N.C., Sr.; Prumo, F.M. A motivational environment for behaviorally deviant junior high school students. *Journal of Abnormal Child Psychology* **1976**, *4*, 263-275, doi:10.1007/BF00917763.
83. Hein, S.; Weeland, J.; Square, A.; Haeffel, G.J.; Chapman, J.; Macomber, D.; Lee, M.; Foley Geib, C.; Grigorenko, E.L. Effectiveness of a social problem solving training in youth in detention or on probation: An RCT and pre-post community implementation. *International Journal of Law and Psychiatry* **2020**, *72*, 101626, doi:10.1016/j.ijlp.2020.101626.
84. Henggeler, S.W.; Melton, G.B.; Smith, L.A. Family preservation using multisystemic therapy: an effective alternative to incarcerating serious juvenile offenders. *Journal of Consulting and Clinical Psychology* **1992**, *60*, 953-961, doi:10.1037//0022-006x.60.6.953.
85. Hentges, R.F.; Weaver Krug, C.M.; Shaw, D.S.; Wilson, M.N.; Dishion, T.J.; Lemery-Chalfant, K. The long-term indirect effect of the early Family Check-Up intervention on adolescent internalizing and externalizing symptoms via inhibitory control. *Development and psychopathology* **2020**, *32*, 1544-1554, doi:10.1017/S0954579419001482.
86. Hilyer, J.C.; Wilson, D.G.; Dillon, C.; Caro, L. Physical Fitness Training and Counseling as Treatment for Youthful Offenders. *Journal of Counseling Psychology* **1982**, *29*, 292-303.
87. Himelstein, S.; Hastings, A.; Shapiro, S.; Heery, M. Mindfulness training for self-regulation and stress with incarcerated youth: A pilot study. *Probation Journal* **2011**, *59*, 151–165.
88. Hobbs, T.R.; Holt, M. The effects of token reinforcement on the behavior of delinquents in cottage settings. *Journal of Applied Behavior Analysis* **1976**, *9*, 189-198, doi:10.1901/jaba.1976.9-189.
89. Hogue, A.; Henderson, C.E.; Schmidt, A.T. Multidimensional Predictors of Treatment Outcome in Usual Care for Adolescent Conduct Problems and Substance Use. *Administration and Policy in Mental Health and Mental Health Services Research* **2017**, *44*, 380-394.

90. Holmqvist, R.; Hill, T.; Lang, A. Effects of Aggression Replacement Training in Young Offender Institutions. *International Journal of Offender Therapy and Comparative Criminology* **2009**, *53*, 74-92.
91. Hoogsteder, L.M.; Stams, G.-J.J.; Schippers, E.E.; Bonnes, D. Responsive Aggression Regulation Therapy (Re-ART): An evaluation study in a Dutch juvenile justice institution in terms of recidivism. *International Journal of Offender Therapy and Comparative Criminology* **2018**, *62*, 4403-4424.
92. Hornsveld, R.H.; Nijman, H.L.; Hollin, C.R.; Kraaimaat, F.W. Aggression control therapy for violent forensic psychiatric patients: Method and clinical practice. *International Journal of Offender Therapy and Comparative Criminology* **2008**, *52*, 222-233.
93. Hornsveld, R.H.J.; Kraaimaat, F.W.; Muris, P.; Zwets, A.J.; Kanters, T. Aggression Replacement Training for Violent Young Men in a Forensic Psychiatric Outpatient Clinic. *Journal of Interpersonal Violence* **2015**, *30*, 3174-3191.
94. Houck, C.D.; Hadley, W.; Barker, D.; Brown, L.K.; Hancock, E.; Almy, B. An Emotion Regulation Intervention to Reduce Risk Behaviors Among at-Risk Early Adolescents. *Prevention Science* **2016**, *17*, 71-82.
95. Hovell, M.F.; Blumberg, E.J.; Liles, S.; Powell, L.; Morrison, T.C.; Duran, G.; Sipan, C.L.; Burkham, S.; Kelley, N. Training AIDS and anger prevention social skills in at-risk adolescents. *Journal of Counseling & Development* **2001**, *79*, 347-355.
96. Huey, S.J., Jr.; Henggeler, S.W.; Brondino, M.J.; Pickrel, S.G. Mechanisms of change in multisystemic therapy: reducing delinquent behavior through therapist adherence and improved family and peer functioning. *Journal of Consulting and Clinical Psychology* **2000**, *68*, 451-467.
97. Huey, W.C.; Rank, R.C. Effects of Counselor and Peer-Led Group Assertive Training on Black Adolescent Aggression. *Journal of Counseling Psychology* **1984**, *31*, 95-98.
98. Jamali, S.; Sabokdast, S.; Nia, H.S.; Goudarzian, A.H.; Beik, S.; Allen, K.A. The effect of life skills training on mental health of Iranian middle school students: a preliminary study. *Iranian journal of psychiatry* **2016**, *11*, 269-273.
99. Jones, C.D.; Lowe, L.A.; Risler, E.A. The Effectiveness of Wilderness Adventure Therapy Programs for Young People Involved in the Juvenile Justice System. *Residential Treatment for Children & Youth* **2004**, *22*, 53-67.

100. Kaffman, M. Hypnosis as an adjunct to psychotherapy in child psychiatry. *Archives of General Psychiatry* **1968**, *18*, 725-738.

101. Kahn, M.W.; McFarland, J. A demographic and treatment evaluation study of institutionalized juvenile offenders. *Journal of Community Psychology* **1973**, *1*, 282-284.

102. Kanchibhotla, D.; Subramanian, S.; Kaushik, B. Association of yogic breathing with perceived stress and conception of strengths and difficulties in teenagers. *Clin Child Psychol Psychiatry* **2021**, 1359104521994633, doi:10.1177/1359104521994633.

103. Kappes, B.M.; Thompson, D.L. Biofeedback vs. video games: effects on impulsivity, locus of control and self-concept with incarcerated juveniles. *Journal of Clinical Psychology* **1985**, *41*, 698-706.

104. Kastner, J.W. Clinical Change in Adolescent Aggressive Behavior: A Group Therapy Approach. *Journal of Child and Adolescent Group Therapy* **1998**, *8*, 23-33.

105. Kauser, R.; Pinquart, M. Effectiveness of an indigenous parent training program on change in parenting styles and delinquent tendencies (challenging behaviors) in Pakistan: A randomized controlled trial. *Journal of Experimental Child Psychology* **2019**, *188*, doi:10.1016/j.jecp.2019.104677.

106. Kaya, F.; Buzlu, S. Effects of Aggression Replacement Training on problem solving, anger and aggressive behaviour among adolescents with criminal attempts in Turkey: A quasi-experimental study. *Archives of Psychiatric Nursing* **2016**, *30*, 729-735.

107. Keiley, M.K. Multiple-family group intervention for incarcerated adolescents and their families: a pilot project. *Journal of Marital and Family Therapy* **2007**, *33*, 106-124.

108. Kellner, M.H.; Bry, B.H. The effects of anger management groups in a day school for emotionally disturbed adolescents. *Adolescence* **1999**, *34*, 645-651.

109. Kellner, M.H.; Bry, B.H.; Colletti, L.-A. Teaching anger management skills to students with severe emotional or behavioral disorders. *Behavioral Disorders* **2002**, *27*, 400-407.

110. Kellner, M.H.; Colletti, L.A.; Bry, B.H. Increasing anger log use during school among middle school students with emotional/behavioral disorders. *Child & Family Behavior Therapy* **2003**, *25*, 7-21.

111. Kethineni, S.; Braithwaite, J. The Effects of a Cognitive- Behavioral Program for At-Risk Youth: Changes in Attitudes, Social Skills, Family, and Community and Peer Relationships. *Victims and Offenders* **2010**, *6*, 93-116.

112. Kifer, R.E.; Lewis, M.A.; Green, D.R.; Phillips, E.L. Training predelinquent youths and their parents to negotiate conflict situations. *Journal of Applied Behavior Analysis* **1974**, *7*, 357-364, doi:10.1901/jaba.1974.7-357.

113. Kimber, B.; Sandell, R.; Bremberg, S. Social and emotional training in Swedish classrooms for the promotion of mental health: results from an effectiveness study in Sweden. *Health Promotion International* **2008**, *23*, 134-143.

114. Langeveld, J.H.; Gundersen, K.K.; Svartdal, F. Social competence as a mediating factor in reduction of behavioral problems. *Scandinavian Journal of Educational Research* **2012**, *56*, 381-399.

115. Larson, J.D. Anger and Aggression Management Techniques through the Think First Curriculum. *Journal of Offender Rehabilitation* **1992**, *18*, 101-117.

116. Larson, J.D.; Calamari, J.E.; West, J.G.; Frevert, T.A. Aggression management with disruptive adolescents in the residential setting: Integration of a cognitive-behavioral component. *Residential Treatment for Children & Youth Violence and Juvenile Justice* **1998**, *15*, 1-9.

117. Lazarus, B.D. Self-Management and Achievement of Students with Behavior Disorders. *Psychology in the Schools* **1993**, *30*, 67-74.

118. Le, T.N.; Proulx, J. Feasibility of Mindfulness-Based Intervention for Incarcerated Mixed-Ethnic Native Hawaiian/Pacific Islander Youth. *Asian American Journal of Psychology* **2015**, *6*, 181-189.

119. Lee, D.Y.; Hallberg, E.T.; Hassard, H. Effects of Assertion Training on Aggressive Behavior of Adolescents. *Journal of Counseling Psychology* **1979**, *26*, 459-461.

120. Leonard, N.R.; Jha, A.P.; Casarjian, B.; Goolsarran, M.; Garcia, C.; Cleland, C.M.; Gwadz, M.V.; Massey, Z. Mindfulness training improves attentional task performance in incarcerated youth: a group randomized controlled intervention trial. *Frontiers in Psychology* **2013**, *4*, 792.

121. Leve, L.D.; Chamberlain, P.; Reid, J.B. Intervention outcomes for girls referred from juvenile justice: effects on delinquency. *Journal of Consulting and Clinical Psychology* **2005**, *73*, 1181-1185, doi:10.1037/0022-006X.73.6.1181.

122. Lök, N.; Bademli, K.; Canbaz, M. The Effects of Anger Management Education on Adolescents' Manner of Displaying Anger and Self-Esteem: A Randomized Controlled Trial. *Archives of Psychiatric Nursing* **2018**, *32*, 75-81.

123. Lombas, A.S.; Jimenez, T.I.; Arguis-Rey, R.; Hernandez-Paniello, S.; Valdivia-Salas, S.; Martin-Albo, J. Impact of the Happy Classrooms Programme on Psychological Well-being, School Aggression, and Classroom Climate. *Mindfulness* **2019**, *10*, 1642-1660.
124. Long, S.J.; Sherer, M. Social Skills Training With Juvenile Offenders. *Child & Family Behavior Therapy* **1984**, *6*, 1-11.
125. Lutz, B. An Institutional Case Study: Emotion Regulation With HeartMath at Santa Cruz County Children's Mental Health. *Global Advances in Health and Medecine* **2014**, *3*, 68-71.
126. MacMahon, J.R.; Gross, R.T. Physical and psychological effects of aerobic exercise in delinquent adolescent males. *American Journal of Diseases of Children* **1988**, *142*, 1361-1366.
127. Marco, J.H.; Garcia-Palacios, A.; Botella, C. Dialectical behavioural therapy for oppositional defiant disorder in adolescents: A case series. *Psicothema* **2013**, *25*, 158-163.
128. Marshall, A.E.; Heward, W.L. Teaching self-management to incarcerated youth. *Behavioral Disorders* **1979**, *4*, 215-226.
129. Martin, G.; Johnson, C.L. The boys totem town neurofeedback project: A pilot study of EEG biofeedback with incarcerated juvenile felons. *Journal of Neurotherapy: Investigations in Neuromodulation, Neurofeedback and Applied Neuroscience* **2005**, *9*, 71-86.
130. Martsch, M.D. A Comparison of Two Group Interventions for Adolescent Aggression: High Process Versus Low Process. *Research on Social Work Practice* **2005**, *15*, 8-18.
131. Marvit, R.C.; Lind, J.; McLaughlin, D.G. Use of videotape to induce attitude change in delinquent adolescents. *American Journal of psychiatry* **1974**, *131*, 996-999.
132. Mason, L.H.; Kubina, R.M.; Valasa, L.L.; Cramer, A.M. Evaluating Effective Writing Instruction for Adolescent Students in an Emotional and Behavior Support Setting. *Behavioral Disorders* **2010**, *35*, 140-156.
133. Mason, W.A.; January, S.A.; Fleming, C.B.; Thompson, R.W.; Parra, G.R.; Haggerty, K.P.; Snyder, J.J. Parent training to reduce problem behaviors over the transition to high school: tests of indirect effects through improved emotion regulation skills. *Children and youth services review* **2016**, *61*, 176-183, doi:10.1016/j.childyouth.2015.12.022.
134. Mathur, S.R.; Rutherford, R.B. Teaching Conversational Social Skills to Delinquent Youth. *Behavioral Disorders* **1994**, *19*, 294-305.

135. Maya, J.; Hidalgo, V.; Jimenez, L.; Lorence, B. Effectiveness of Scene-Based Psychodramatic Family Therapy (SB-PFT) in adolescents with behavioural problems. *Health Soc Care Community* **2019**, *28*, 555-567, doi:10.1111/hsc.12888.
136. Mazerolle, L.; Antrobus, E.; Bennett, S.; Eggins, E. Reducing Truancy and Fostering a Willingness to Attend School: Results from a Randomized Trial of a Police-School Partnership Program. *Prevention science* **2017**, *18*, 469-480, doi:10.1007/s11121-017-0771-7.
137. Mazerolle, L.; Bennett, S.; Antrobus, E.; Cardwell, S.M.; Eggins, E.; Piquero, A.R. Disrupting the Pathway from Truancy to Delinquency: A Randomized Field Trial Test of the Longitudinal Impact of a School Engagement Program. *Journal of Quantitative Criminology* **2019**, *35*, 663-689.
138. Mazerolle, L.; Bennett, S.; Antrobus, E.; Eggins, E. The Coproduction of Truancy Control: Results from a Randomized Trial of a Police–Schools Partnership Program. *Journal of Research in Crime and Delinquency* **2017**, *54*, 791-823.
139. Mazerolle, L.; Cardwell, S.M.; Antrobus, E.; Piquero, A.R. Policing partnerships to address youth antisocial behavior: How parental risk-taking shapes child outcomes. *Criminol Public Pol* **2020**, *19*, 883-904, doi:10.1111/1745-9133.12510.
140. McAllister, L.W.; Stachowiak, J.G.; Baer, D.M.; Conderman, L. The application of operant conditioning techniques in a secondary school classroom. *Journal of Applied Behavior Analysis* **1969**, *2*, 277-285, doi:10.1901/jaba.1969.2-277.
141. McCarthy-Tucker, S.; Gold, A.; Garcia, E., III. Effects of anger management training on aggressive behavior in adolescent boys. *Journal of Offender Rehabilitation* **1999**, *29*, 129-141.
142. McCraty, R.; Atkinson, M.; Tomasino, D.; Goelitz, J.; Mayrovitz, H.N. The impact of an emotional self-management skills course on psychosocial functioning and autonomic recovery to stress in middle school children. *Integrative Physiological and Behavioral Science* **1999**, *34*, 246-268.
143. McCullough, J.P.; Huntsinger, G.M.; Nay, W.R. Self-control treatment of aggression in a 16-year-old male. *Journal of Consulting and Clinical Psychology* **1977**, *45*, 322-331.
144. McMahon, S.D.; Washburn, J.J. Violence Prevention: An Evaluation of Program Effects with Urban African American Students. *The Journal of Primary Prevention* **2003**, *24*, 43-62.

145. McNamara, E.; Heard, C. Self-control through self-recording. *Special Education Forward Trends* **1976**, *3*, 21-23.

146. McNamara, E.; Jolly, M. The reduction of disruptive behaviour using feedback on-task behaviour: an across setting study of a class of 12- and 13-year-old pupils. *Behavioural Psychotherapy* **1990**, *18*, 103-119.

147. McWhirter, B.T.; Page, G.L. Effects of anger management and goal setting group interventions on state-trait anger and self-efficacy beliefs among high risk adolescents. *Current Psychology* **1999**, *18*, 223-237.

148. Minkos, M.L.; Chafouleas, S.M.; Bray, M.A.; LaSalle, T.P. Brief Report: A Preliminary Investigation of a Mindful Breathing Intervention to Increase Academic Engagement in an Alternative Educational Setting. *Behavioral Disorders* **2018**, *43*, 436-443.

149. Minor, K.I.; Elrod, P. The effects of a probation intervention on juvenile offenders' self-concepts, loci of control, and perceptions of juvenil justice. *Youth & Society* **1994**, *25*, 490-511.

150. Mohammadi, M.R.; Salmanian, M.; Ghobari-Bonab, B.; Bolhari, J. Spiritual psychotherapy for adolescents with conduct disorder: Designing and piloting a therapeutic package. *Iranian Journal of Psychiatry* **2017**, *12*, 255-261.

151. Moneta, I.; Rousseau, C. Emotional expression and regulation in a school-based drama workshop for immigrant adolescents with behavioral and learning difficulties. *The Arts in Psychotherapy* **2008**, *35*, 329-340.

152. Moretti, M.M.; Obsuth, I. Effectiveness of an attachment-focused manualized intervention for parents of teens at risk for aggressive behaviour: The Connect Program. *Journal of Adolescence* **2009**, *32*, 1347-1357.

153. Mutiso, V.; Tele, A.; Musyimi, C.; Gitonga, I.; Musau, A.; Ndetei, D. Effectiveness of life skills education and psychoeducation on emotional and behavioral problems among adolescents in institutional care in Kenya: a longitudinal study. *Child and Adolescent Mental Health* **2018**, *23*, 351-358, doi:10.1111/camh.12232.

154. Neel, R.S.; De Bruler, L. The effects of self-management of school attendance by problem adolescents. *Adolescence* **1979**, *14*, 175-184.

155. Nelson-Gray, R.O.; Keane, S.P.; Hurst, R.M.; Mitchell, J.T.; Warburton, J.B.; Chok, J.T.; Cobb, A.R. A modified DBT skills training program for oppositional defiant adolescents: promising preliminary findings. *Behaviour Research and Therapy* **2006**, *44*, 1811-1820, doi:10.1016/j.brat.2006.01.004.

156. Nickel, C.; Lahmann, C.; Tritt, K.; Loew, T.H.; Rother, W.K.; Nickel, M.K. Short Communication: Stressed aggressive adolescents benefit from progressive muscle relaxation: A random, prospective, controlled trial. *Stress and Health* **2005**, *21*, 169-175.
157. Niles, W.J. Effects of a Moral Development Discussion Group on Delinquent and Predelinquent Boys. *Journal of Conseling Psychology* **1986**, *33*, 45-51.
158. Ninness, H.A.; Ellis, J.; Miller, W.B.; Baker, D.; Rutherford, R. The effect of a self-management training package on the transfer of aggression control procedures in the absence of supervision. *Behavior modification* **1995**, *19*, 464-490.
159. Nugent, W.R.; Bruley, C.; Allen, P. The effects of Aggression Replacement Training on male and female antisocial behavior in a runaway shelter. *Research on Social Work Practice* **1999**, *9*, 466-482.
160. Nugent, W.R.; Champlin, D.; Wiinimaki, L. The effects of anger control training on adolescent antisocial behavior. *Research on Social Work Practrice* **1997**, *7*, 446-462.
161. Ollendick, T.H.; Hersen, M. Social skills training for juvenile delinquents. *Behaviour Research and Therapy* **1979**, *17*, 547-554, doi:10.1016/0005-7967(79)90098-6.
162. Oruche, U.M.; Robb, S.L.; Draucker, C.B.; Aalsma, M.; Pescosolido, B.; Chacko, A.; Ofner, S.; Bakoyannis, G.; Brown-Podgorski, B. Pilot Randomized Trial of a Family Management Efficacy Intervention for Caregivers of African American Adolescents with Disruptive Behaviors. *Child & Youth Care Forum* **2018**, *47*, 803-827.
163. Panosky, D.M.; Shelton, D. Evaluating an Adolescent Behavioral Program: Leadership, Education, Achievement, and Development for Adolescent Female Offenders in Corrections. *International Association of Forensic Nurses* **2015**, *11*, 144-153.
164. Pardo, E.S.; Rivas, A.F.; Barnier, P.O.; Mirabent, M.B.; Lizeaga, I.K.; Cosgaya, A.D.; Alcantara, A.C.; Gonzalez, E.V.; Aguirre, B.; Torres, M.A.G. A qualitative research of adolescents with behavioral problems about their experience in a dialectical behavior therapy skills training group. *BMC Psychiatry* **2020**, *20*, 245, doi:10.1186/s12888-020-02649-2.
165. Patrick, J.; Rich, C. Anger Management Taught to Adolescents with an Experiential Object Relations Approach. *Child and Adolescent Social Work Journal* **2004**, *21*, 85-100.
166. Patterson, G.R. Interventions for boys with conduct problems: multiple settings, treatments, and criteria. *Journal of Consulting and Clinical Psychology* **1974**, *42*, 471-481, doi:10.1037/h0036731.

167. Pazaratz, D. Intervention strategies with a behavior disordered male. *Residential Treatment for Children & Youth* **1998**, *16*, 1-12.
168. Peterson, L., D.,; Young, K.; West, R.P.; Peterson, M.H. Effects of student self-management on generalization of student performance to regular classrooms. *Education and Treatment of Children* **1999**, *22*, 357-372.
169. Phillips, E.L.; Phillips, E.A.; Fixsen, D.L.; Wolf, M.M. Achievment place: modification of the behaviors of pre-delinquent boys within a token economy. *Journal of Applied Behavior Analysis* **1971**, *4*, 45-59.
170. Pop-Jordanova, N. Heart Rate Variability in the Assessment and Biofeedback Training of Common Mental Health Problems in Children. *Medical Archives* **2009**, *63*, 248-252.
171. Presley, J.A.; Hughes, C. Peers as teachers of anger management to high school students with behavioral disorders. *Behavioral Disorders* **2000**, *25*, 114-130.
172. Puskar, K.R.; Ren, D.X.; McFadden, T. Testing the 'Teaching Kids to Cope with Anger' Youth Anger Intervention Program in a Rural School-based Sample. *Issues in Mental Health Nursing* **2015**, *36*, 200-208.
173. Quinn, W.H.; Van Dyke, D.J. A multiple family group intervention for first-time juvenile offenders: comparisons with probation and dropouts on recidivism. *Journal of Community Psychology* **2004**, *32*, 177-200.
174. Ramadoss, R.; Bose, B.K. Transformative Life Skills: Pilot Studies of a Yoga Model for Reducing Perceived Stress and Improving Self-Control in Vulnerable Youth. *International Journal of Yoga Therapy* **2010**, *20*, 75-80.
175. Rapp-Paglicci, L.; Stewart, C.; Rowe, W. Can a Self-Regulation Skills and Cultural Arts Program Promote Positive Outcomes in Mental Health Symptoms and Academic Achievement for At-Risk Youth? *Journal of Social Service Research* **2011**, *37*, 309-319.
176. Redondo, S.; Martinez-Catena, A.; Andres-Pueyo, A. Therapeutic Effects of a Cognitive-Behavioural Treatment with Juvenile Offenders. *The European Journal of Psychology Applied to Legal Context* **2012**, *4*, 159-178.
177. Richman, L.C.; Harper, D.C. Parental child-rearing characteristics and delinquent adolescents' response to behavioral treatment. *American Journal of Orthopsychiatry* **1979**, *49*, 527-529.
178. Rickson, D.J.; Watkins, W.G. Music therapy to promote prosocial behaviors in aggressive adolescent boys--a pilot study. *Journal of Music Therapy* **2003**, *40*, 283-301, doi:10.1093/jmt/40.4.283.

179. Rizzo, C.J.; Houck, C.; Barker, D.; Collibee, C.; Hood, E.; Bala, K. Project STRONG: an Online, Parent-Son Intervention for the Prevention of Dating Violence among Early Adolescent Boys. *Prevention Science* **2020**, doi:10.1007/s11121-020-01168-6.
180. Robinson, T.R.; Smith, S.W.; Miller, M.D. Effect of a Cognitive-Behavioral Intervention on Responses to Anger by Middle School Students with Chronic Behavior Problems. *Behavioral Disorders* **2002**, *27*, 256-271.
181. Rodriguez, A.; Esquivel, M.J.; Rodriguez, H.; Fonseca, H. Effect of a Program of Sport-Recreational Activities on Aggression and Prosocial Values among Youth Living in Social Risk. *European Journal of Human Movement* **2016**, *37*, 143-162.
182. Rogevich, M.E.; Perin, D. Effects on science summarization of a reading comprehension intervention for adolescents with behavior and attention disorders. *Exceptional Children* **2008**, *74*, 135-154.
183. Rohde, P.; Jorgensen, J.S.; Seeley, J.R.; Mace, D.E. Pilot Evaluation of the Coping Course: A Cognitive-Behavioral Intervention to Enhance Coping Skills in Incarcerated Youth. *Journal of the American Academy of Child & Adolescent Psychiatry* **2004**, *43*, 669-676.
184. Ronen, T. Imparting Self-Control Skills to Decrease Aggressive Behavior in a 12-Year-Old Boy. *Journal of Social Work* **2004**, *4*, 269–288.
185. Ronen, T.; Rosenbaum, M. Developing learned resourcefulness in adolescents to help them reduce their aggressive behavior: Preliminary findings. *Research on Social Work Practice* **2009**, *20*, 410-426.
186. Rosenberg, S. Inoculation Effect in Prevention of Increased Verbal Aggression in Schools. *Psychological Reports* **2004**, *95*, 1219-1226.
187. Rowlands, A.; Fisher, M.; Mishra, J.; Nahum, M.; Brandrett, B.; Reinke, M.; Caldwell, M.; Kiehl, K.A.; Vinogradov, S. Cognitive Training for Very High Risk Incarcerated Adolescent Males. *Frontiers in Psychiatry* **2020**, *11*, doi:10.3389/fpsyt.2020.00225.
188. Ruttledge, R.A.; Petrides, K.V. A cognitive behavioural group approach for adolescents with disruptive behaviour in schools. *School Psychology International* **2011**, *33*, 223-239.
189. Santogrossi, D.A.; O'Leary K, D.; Romanczyk, R.G.; Kaufman, K.F. Self-evaluation by adolescents in a psychiatric hospital school token program. *Journal of Applied Behavior Analysis* **1973**, *6*, 277-287, doi:10.1901/jaba.1973.6-277.

190. Sanz de Acedo Lizarraga, M.L.; Iriarte Iriarte, M.D. Enhancement of cognitive functioning and self-regulation of learning in adolescents. *The Spanish Journal of Psychology* **2001**, *4*, 55-64.
191. Scherer, D.G.; Brondino, M.J.; Henggeler, S.W.; Melton, G.B.; H., H.J.-. Multisystemic Family Preservation Therapy: Preliminary Findings From a Study of Rural and Minority Serious Adolescent Offenders. *Journal of emotional and behavioral disorders* **1994**, *2*, 198-206.
192. Schippers, E.E.; Hoogsteder, L.M.; Stams, G. Responsive Aggression Regulation Therapy (Re-ART) Improves Executive Functioning in Adolescents and Young Adults with Severe Aggression Problems: A Pilot Study. *Journal of Forensic Sciences* **2020**, doi:10.1111/1556-4029.14539.
193. Schlichter, K.J.; Horan, J.J. Effects of Stress Inoculation on the Anger and Aggression Management Skills of Institutionalized Juvenile Delinquents. *Cognitive Therapy and Research* **1981**, *5*, 359-365.
194. Schloss, P.J.; Smith, M.; Smaldino, S.; Field, M.; Tiffin, R., & ; Ramsey, D. Social learning treatment of self-injurious and aggressive behaviors of a hearing impaired youth. *Journal of the American Deafness and Rehabilitation Association* **1983**, *17*, 16-22.
195. Schuurmans, A.A.T.; Nijhof, K.S.; Engels, R.C.M.E.; Granic, I. Using a Videogame Intervention to Reduce Anxiety and Externalizing Problems among Youths in Residential Care: an Initial Randomized Controlled Trial. *Journal of Psychopathology and Behavioral Assessment* **2018**, *40*, 344-354.
196. Seckman, A.; Paun, O.; Heipp, B.; Van Stee, M.; Keels-Lowe, V.; Beel, F.; Spoon, C.; Fogg, L.; Delaney, K.R. Evaluation of the use of a sensory room on an adolescent inpatient unit and its impact on restraint and seclusion prevention. *Journal of Child and Adolescent Psychiatric Nursing* **2016**, *30*, 90-97.
197. Seivert, N.P.; Cano, A.; Casey, R.J.; May, D.K.; Johnson, A. Animal Assisted Therapy for Incarcerated Youth: A Randomized Controlled Trial. *Applied developmental science* **2018**, *22*, 139-153.
198. Shahbazi, M.; Ghanbari, F.; Jafarinasab, A.; Vaziri, S.M.; Foji, S.; Rahimi, Z.; Hasan-Nezhad, F.; Goudarzian, M. The Effectiveness of Anger Management's Training on Difficulty of Adolescent's Emotion Regulation. *Journal of Fundamental and Applied Sciences* **2017**, *9*, 879-888.

199. Sharry, J.; Owens, C. 'The rules of engagement': A case study of a group with 'angry' adolescents. *Clinical Child Psychology and Psychiatry* **2000**, *5*, 53-62.

200. Shivrattan, J.L. Social Interactional Training and Incarcerated Juvenile Delinquents. *Canadian Journal of Criminology* **1988**, *30*, 145-164.

201. Singh, N.N.; Lancioni, G.E.; Singh Joy, S.D.; Winton, A.S.W.; Sabaawi, M.; Wahler, R.G.; J., S. Adolescents With Conduct Disorder Can Be Mindful of Their Aggressive Behavior. *Journal of Emotional and Behavioral Disorders* **2007**, *15*, 56-63.

202. Smaller, M.D. Psychoanalysis and the Forward Edge Hit the Streets: The Analytic Service to Adolescents Program (ASAP). *Psychoanalytic Inquiry* **2012**, *32*, 136-146.

203. Smith, D.J.; Young, K.R.; West, R.P.; Morgan, D.P.; Rhode, G. Reducing the Disruptive Behavior of Junior High School Students: A Classroom Self-Management Procedure. *Behavioral Disorders* **1988**, *13*, 231-239.

204. Smith, S.W.; Daunic, A.P.; Algina, J.; Pitts, D.L.; Merrill, K.L.; Cumming, M.M.; Allen, C. Self-regulation for students with emotional and behavioral disorders: Preliminary effects of the I Control curriculum. *Journal of Emotional and Behavioral Disorders* **2017**, *25*, 143-156.

205. Snyder, E.P.; Shapiro, E.S. Teaching Students with Emotional/Behavioral Disorders the Skills to Participate in the Development of Their Own IEPs. *Behavioral Disorders* **1997**, *22*, 246-259.

206. Snyder, J.J.; White, M.J. The use of cognitive self-instruction in the treatment of behaviorally disturbed adolescents. *Behavior therapy* **1979**, *10*, 227-235.

207. Snyder, K.V.; Kymissis, P.; Kessler, K. Anger management for adolescents: Efficacy of brief group therapy. *Journal of the American Academy of Child & Adolescent Psychiatry* **1999**, *38*, 1409-1416.

208. Sosa-Rubi, S.G.; Saavedra-Avendano, B.; Piras, C.; Van Buren, S.J.; Bautista-Arredondo, S. True Love: Effectiveness of a School-Based Program to Reduce Dating Violence Among Adolescents in Mexico City. *Prevention Science* **2017**, *18*, 804-817.

209. Spence, S.H.; Marzillier, J.S. Social skills training with adolescent male offenders - II. short-term, long-term and generalized effects. *Behaviour Research and Therapy* **1981**, *19*, 349-368.

210. Stormshak, E.A.; Dishion, T.J.; Light, J.; Yasui, M. Implementing family-centered interventions within the public middle school: linking service delivery to change in

student problem behavior. *Journal of Abnormal Child Psychology* **2005**, *33*, 723-733, doi:10.1007/s10802-005-7650-6.

211. Sutton, D.; Wilson, M.; Van Kessel, K.; Vanderpyl, J. Optimizing arousal to manage aggression: a pilot study of sensory modulation. *International Journal of Mental Health Nursing* **2013**, *22*, 500-511.
212. Syzmanski, T.; Casey, R.J.; Johnson, A.; Cano, A.; Albright, D.; Seivert, N.P. Dog Training Intervention Shows Social-Cognitive Change in the Journals of Incarcerated Youth. *Frontiers in Veterinary Science* **2018**, *5*.
213. Teeter, P.A.; Rumsey, R.; Natoli, L.; Naylor, D.; Smith, R. Therapeutic interventions to increase social competence in teens with impulse control deficits. *Journal of Psychotherapy in Independent Practice* **2000**, *1*, 49-70.
214. Thoder, V.J.; Cautilli, J.D. An Independent Evaluation of Mode Deactivation Therapy for Juvenile Offenders. *The International Journal of Behavioral Consultation and Therapy* **2011**, *7*, 41–46.
215. Titilayo, H.; Aderanti, R.A. Differential effectiveness of self-management and token reinforcement in the treatment of adolescents' disorderliness. *IFE PsychologIA: An International Journal* **2012**, *20*, 134-151.
216. Trajkovic, N.; Pajek, M.; Sporis, G.; Petrinovic, L.; Bogataj, S. Reducing Aggression and Improving Physical Fitness in Adolescents Through an After-School Volleyball Program. *Frontiers in psychology* **2020**, *11*, 2081, doi:10.3389/fpsyg.2020.02081.
217. Trulson, M.E. Martial Arts Training: A Novel "Cure" for Juvenile Delinquency *Human Relations* **1986**, *39*, 1131-1140.
218. Trupin, E.J.; Kerns, S.E.U.; Walker, S.C.; DeRobertis, M.T.; Stewart, D.G. Family Integrated Transitions: A Promising Program for Juvenile Offenders with Co-Occurring Disorders. *Journal of Child & Adolescent Substance Abuse* **2011**, *20*, 421-436.
219. Twemlow, S.W.; Sacco, F.C. The application of traditional martial arts practice and theory to the treatment of violent adolescents. *Adolescence* **1998**, *33*, 505-518.
220. Tyson, E.H. Hip Hop Therapy: An Exploratory Study of a Rap Music Intervention with At-Risk and Delinquent Youth. *Journal of Poetry Therapy* **2002**, *15*, 131-144.
221. Umbach, R.; Raine, A.; Leonard, N.R. Cognitive Decline as a Result of Incarceration and the Effects of a CBT/MT Intervention: A Cluster-Randomized Controlled Trial. *Criminal Justice & Behavior* **2018**, *45*, 31-55.

222. Uzunoglu, G.; Baysan Arabaci, L. The effect of psychoeducation on the anger management of adolescents diagnosed with conduct disorder. *Dusunen Adam The Journal of Psychiatry and Neurological Sciences* **2017**, *30*, 344-353.
223. Valliant, P.M.; Jensen, B.; Ravenbrook, L. Brief Cognitive-Behavioral Therapy with Male-Adolescent Offenders in Open Custody or on Probation - an Evaluation of Management of Anger. *Psychological Reports* **1995**, *76*, 1056-1058.
224. Van Bockstaele, B.; van der Molen, M.J.; van Nieuwenhuijzen, M.; Salemink, E. Modification of hostile attribution bias reduces self-reported reactive aggressive behavior in adolescents. *Journal of experimental child psychology* **2020**, *194*, 104811, doi:10.1016/j.jecp.2020.104811.
225. Van Manen, T.G.; Prins, P.J.; Emmelkamp, P.M. Reducing aggressive behavior in boys with a social cognitive group treatment: results of a randomized, controlled trial. *Journal of the American Academy of Child & Adolescent Psychiatry* **2004**, *43*, 1478-1487.
226. Van Vliet, K.J.; Foskett, A.J.; Williams, J.L.; Singhal, A.; Dolcos, F.; Vohra, S. Impact of a mindfulness-based stress reduction program from the perspective of adolescents with serious mental health concerns. *Child and Adolescent Mental Health* **2017**, *22*, 16-22.
227. Van Wijk-Herbrink, M.F.; Broers, N.J.; Roelofs, J.; Bernstein, D.P. Schema Therapy in Adolescents with Disruptive Behavior Disorders. *International Journal of Forensic Mental Health* **2017**, *16*, 261-279.
228. Wainryb, C.; Pasupathi, M.; Bourne, S.; Oldroyd, K. Stories for all ages: Narrating anger reduces distress across childhood and adolescence. *Developmental Psychology* **2018**, *54*, 1072-1085.
229. West, M.; Melvin, G.; McNamara, F.; Gordon, M. An evaluation of the use and efficacy of a sensory room within an adolescent psychiatric inpatient unit. *Australian Occupational Therapy Journal* **2017**, *64*, 253-263.
230. Whitfield, G.W. Validating school social work: An evaluation of a cognitive-behavioral approach to reduce school violence. *Research on Social Work Practice* **1999**, *9*, 399-426.
231. Williams, D.Y.; Akamatsu, T. Cognitive self-guidance training with juvenile delinquents: Applicability and generalization. *Cognitive Therapy and Research,* **1978**, *2*, 285-288.
232. Wodarski, J.S.; Filipczak, J.; McCombs, D.; Koustenis, G.; Rusilko, S. Follow-up on behavioral intervention with troublesome adolescents. *Journal of Behavior Therapy and Experimental Psychiatry* **1979**, *10*, 181-188.

233. Wolff, J.C.; Ollendick, T.H. Treatment of comorbid conduct problems and depression in youth: A pilot study. *Child & Family Behavior Therapy* **2012**, *34*, 141-155.
234. Wongtongkam, N.; Ward, P.R.; Day, A.; Winefield, A.H. A trial of mindfulness meditation to reduce anger and violence in Thai youth. *International Journal of Mental Health and Addiction* **2014**, *12*, 169-180.
235. Yektatalab, S.; Khodadadi, S.; Moattari, M.; Hosseiny, F.N.; Zare, N. A web-based anger management program for parent-female adolescents' conflicts: a cluster randomized controlled trial. *International Journal of Adolescent Medicine and Health* **2017**, *31*, doi:10.1515/ijamh-2017-0012.
236. Zapolski, T.C.B.; Smith, G.T. Pilot Study: Implementing a Brief DBT Skills Program in Schools to Reduce Health Risk Behaviors Among Early Adolescents. *The Journal of School Nursing* **2017**, *33*, 198-204.
237. Zhou, Y.Q.; Gan, D.Z.Q.; Hoo, E.C.C.; Chong, D.; Chu, C.M. Evaluating the Violence Prevention Program: group and individual changes in aggression, anger, self-control, and empathy. *The Journal of Forensic Psychiatry & Psychology* **2018**, *29*, 265-287.
238. Zivin, G.; Hassan, N.R.; DePaula, G.F.; Monti, D.A.; Harlan, C.; Hossain, K.D.; Patterson, K. An effective approach to violence prevention: traditional martial arts in middle school. *Adolescence* **2001**, *36*, 443-459.
