# Supplementary material for: Non-Pharmaceutical Interventions for Self-Regulatory Failures in Adolescents Suffering from Externalizing Symptoms: A Scoping Review
Source: Biomedicines. 2021 Aug 24;9(9):1081. doi: 10.3390/biomedicines9091081 (PMC8466021; doi:10.3390/biomedicines9091081)
Supplement: Supplementary file 1 [file biomedicines-09-01081-s001.zip › Supplementary_file_3.pdf]

Supplementary file 3: Non-pharmaceutical interventions for self-regulatory failures in adolescents suffering from externalizing symptoms: A scoping review

Supplementary Table S2. Definition of the categories

Concept	Subcategories	Definition
**Sample**	Universal prevention	This prevention includes school-based intervention (e.g., truancy reduction program) and targets healthy adolescents.
	Selective prevention	Targets adolescents who are at risk of embarking on a criminal career (e.g., concerns adolescents with emotional or behavioral difficulties, student considered disruptive by the teacher or with academic difficulties).
	Indicated prevention	Is subsequent to the commission of an offence and concerns youths incarcerated in a juvenile facility or under probationary period (i.e., sentence that allows adolescents offenders to remain with their family while under the supervision of the court).
	Treatment	Concerns adolescents with severe externalizing disorders
**Self-regulation**	Cognition	Cognitive self-regulation entails acting in accordance with values and to adopt long term goals. This category is closely related to self control skills, self management or self esteem.
	Emotion	Linked to the control of emotions, particularly to anger management and arousal (emotional state).
	Social	Concerns the social adaptation and social competence (e.g., ability to improve family and friendship relationship).
	Physiology	Refers to capacity to regulate the bodily process during a stressful situation (e.g., survival strategies facing a challenging situation or ability to regulate heart rate).
**Externalizing symptoms**	Aggressiveness	Refers to hostility and inappropriate interactions with peers and adults (e.g., yelling inside of the classroom, interrupting others, truancy).
	Irritability / anger	Linked to anger dysregulation (e.g., irritable mood, angry outburst in a specific situation).
	Conduct problems	Characterized by breaking rules and violation of social norms (e.g., disturbing disruptive behaviors).
	Impulsivity	Refers to an impulse control disorder and to the tendency to seek highly stimulating experiences (i.e., engaging in a risky activity without considering negative consequences, with a lack of premeditation or risk taking.
	Offending	Related to illegal and criminal activities (e.g., violent crime, violation of societal norms) and criminal conduct.
	Attention-deficit/hyperactivity disorder (ADHD)	Refers to ADHD symptoms such as inattention, excessive physical movement, acting without thinking
	Substance use	Refers to drugs and alcohol abuse (e.g., tobacco, alcohol, cannabis).
	Sexual risky behaviors	Concerns unprotected sexual activity or having more than one sexual partner, but not criminal sexual offence (i.e., sexual assault).

Supplementary file 3: Non-pharmaceutical interventions for self-regulatory failures in adolescents suffering from externalizing symptoms: A scoping review

**Internalizing symptoms**	Anxiety	Characterized by feelings of tension or worried thoughts (e.g., apprehension, fear).
	Depressive symptoms	Emotional state marked by negative feelings such as excessive sadness or pessimism.
	Somatization	Refers to the expression of psychological distress as somatic symptoms (e.g., chronic pain).
	Self-injurious behaviors	Refers to commission of deliberate injury to one's own body (e.g., non-suicidal self-injury behaviors).
**Interventions**	Body-based interventions	Describe fitness and health interventions in order to improve physical activity and well-being (e.g., yoga, massage, martial art, cardiovascular physical fitness).
	Emotional-based interventions	Targeting specifically anger management therapy in order to control emotion and enhance frustration tolerance (e.g., Juvenile Justice Anger Management).
	Cognitive-based interventions	Concern approach that make use of both cognitive and behavioral tools (e.g., problem-solving training, Self-Regulated Strategy Development).
	Mind-based interventions	Aim to foster attention on what is happening in present situation, in order to improve mood, increase compassion for others and to train individuals to meditation exercises and to practice it in daily life (e.g., mindfulness, meditative martial art).
	Curriculum-based interventions	Are multiple stepped therapy targeting several concepts, such emotion (e.g., anger control), cognition (e.g., self-management to resolve a problem) and social (e.g., friendship skills).
	Family-based interventions	Therapies or interventions that involve both adolescent and his parents/caregivers, in order to improve communication or cohesion within family (e.g., parent management training, program of skills building of parents and youths).
	Social-based interventions	Target social skills in order to have appropriate interactions with peers and adults (e.g., conflict resolution, ability school engagement, social problem solving skills).
	Externally mediated interventions	Concern therapies with an external media to act on disruptive behavior (e.g., music therapy, sensory room in hospital, animal assisted therapy).
